# Metagenomic insights into mangrove lignocellulolytic bacteria and functional analysis of a glucose-tolerant GH 1 β-glucosidase

**DOI:** 10.1007/s13205-026-04788-x

**Published:** 2026-04-15

**Authors:** Kheng Loong Chong, Kok Jun Liew, Faezah Mohd Salleh, Chun Shiong Chong

**Affiliations:** Department of Biosciences, Faculty of Science, Universiti Teknologi Malaysia, 81310 Skudai, Johor Malaysia

**Keywords:** Bacterial communities, Lignocellulose biodegradation, Mangrove, Metagenomics, Taxonomy and functional profiling

## Abstract

Mangrove ecosystems contain abundant lignocellulosic biomass and mangrove microorganisms that are capable of degrading plant polymers. In this study, a shotgun metagenomic approach was employed to explore the bacterial communities from Tanjung Piai National Park, Malaysia and their genes involved in lignocellulosic biomass degradation. A total of 148 of carbohydrate active enzymes (CAZy) genes spanning GH, CE, and AA families were identified with lignocellulolytic abilities. These enzymes included 20 cellulases, 46 hemicellulases, and 82 lignin-modifying enzymes. Approximately 89.19% of these genes were found from underexplored bacterial lineages. A set of lignocellulolytic genes derived from diverse bacterial taxa highlighted the synergistic action of mangrove bacteria in lignocellulose degradation. To validate the functionality of these genetic resources, one of the genes (*BGL3_GH1*) encoding a β-glucosidase was selected for expression and characterisation. The recombinant enzyme showed optimal activity at 60 ℃ and pH 7, retained up to 75% activity at 10% (w/v) NaCl. The enzyme exhibited a 1.6 to 2.1-fold in enzyme activity with glucose concentration up to 2 M. In a two-step saccharification assay using sugarcane bagasse, supplementation with recombinant BGL3_GH1 enhanced the saccharification yield (0.0674 g g^− 1^ biomass) compared with treatments using commercial cellulase or recombinant BGL3_GH1 alone. These findings reveal the functional diversity of lignocellulose-degrading genes in mangrove bacteria and identify recombinant BGL3_GH1 as a potential enzyme candidate for biomass conversion application.

**Supplementary Information:**

The online version contains supplementary material available at 10.1007/s13205-026-04788-x.

## Introduction

Mangroves are among the most carbon-rich ecosystems in the tropics, where fallen leaves, twigs, roots, and woody debris contribute to the organic matter pool (Priya et al. 2018). Plant-derived organic matter in mangroves is primarily composed of lignocellulose, a structural component of plant cell walls made up of cellulose, hemicellulose, and lignin (Zhang et al. 2021). The abundance of lignocellulosic material in mangrove ecosystem exerts selective pressure on indigenous microorganisms driving the evolution of genes and enzymes specialized for polymer degradation (Kathiresan 2019). The degradation of lignocellulose releases simple sugar molecules such as glucose, mannose, and xylose that serve as key energy source to support microbial growth and survival (Mai et al. 2021; Yousuf et al. 2020).

Lignocellulose degradation is mediated by carbohydrate-active enzymes (CAZymes), including cellulases, hemicellulases, lytic polysaccharide monooxygenases (LPMOs), and lignin-modifying enzymes (Zhai et al. 2022). CAZymes were categorized into six major domains: glycoside hydrolases (GHs), glycosyltransferases (GTs), polysaccharide lyases (PLs), carbohydrate esterases (CEs), auxiliary activities (AA), and carbohydrate-binding modules (CBMs) (Terrapon et al. 2017). Among these, GH, CE, and AA families are particularly important for lignocellulose degradation (Bredon et al. 2018; Liew et al. 2022; Xiao et al. 2022). For instance, cellulases and hemicellulases are classified under GH and CE families, while LPMO and lignin-modifying enzymes fall within the AA family (Grgas et al. 2023; Gunjal et al. 2020).

Within the cellulase system, β-glucosidase (EC 3.2.1.21) performs the rate-limiting step of cellulose degradation by hydrolysing cellobiose to glucose (Sengupta et al. 2023). This reaction is crucial for efficient biomass saccharification, which converts lignocellulose into fermentable sugars for biofuel production (Sharma et al. 2022). However, β-glucosidases are often inhibited by accumulated glucose, reducing overall hydrolysis efficiency (Sengupta et al. 2023). Therefore, discovering new β-glucosidases with high glucose tolerance is essential to improve industrial biomass conversion (Mujtaba et al. 2023).

Although numerous lignocellulolytic enzymes have been discovered through culture-dependent methods, a substantial proportion of microorganisms with lignocellulolytic potential remain unexplored. This is due to the limitations of culture-based techniques, as only approximately 1% of bacterial species are cultivated under laboratory conditions (Bodor et al. 2020). Metagenomics provides a culture-independent approach to analyse genetic material directly from environmental samples using bioinformatics tools and reference databases (CAZy, eggNOG, KEGG, and NCBI). This approach allows access to previously overlooked bacterial communities and facilitates the identification of their functional traits and ecological roles (Sagar et al. 2024). Through metagenomics, novel lignocellulolytic gene pools have been uncovered from diverse environments, offering a valuable reservoir of enzymes with potential applications in various biotechnological processes (Liew et al. 2022; Rubio-Portillo et al. 2021; Zhao et al. 2019).

Despite Malaysia ranks among the top 10 countries with the largest mangrove coverage, metagenomics studies focusing specifically on lignocellulolytic genes in Malaysia mangrove soils remain limited (Priyashantha and Taufikurahman 2020). In the present study, shotgun metagenomic sequencing was employed to investigate lignocellulose-degrading gene profiles from the Tanjung Piai mangrove soil microbiome. The aims of this study were: (1) to profile the functional genes of mangrove microbiome; (2) to characterize the contribution of bacterial communities to lignocellulose degradation; and (3) to clone, express, and biochemically characterize a GH1 β-glucosidase (BGL3_GH1) to determine its potential in biomass saccharification.

## Materials and methods

### Sample collection and soil physiochemical parameters

**Fig. 1:**
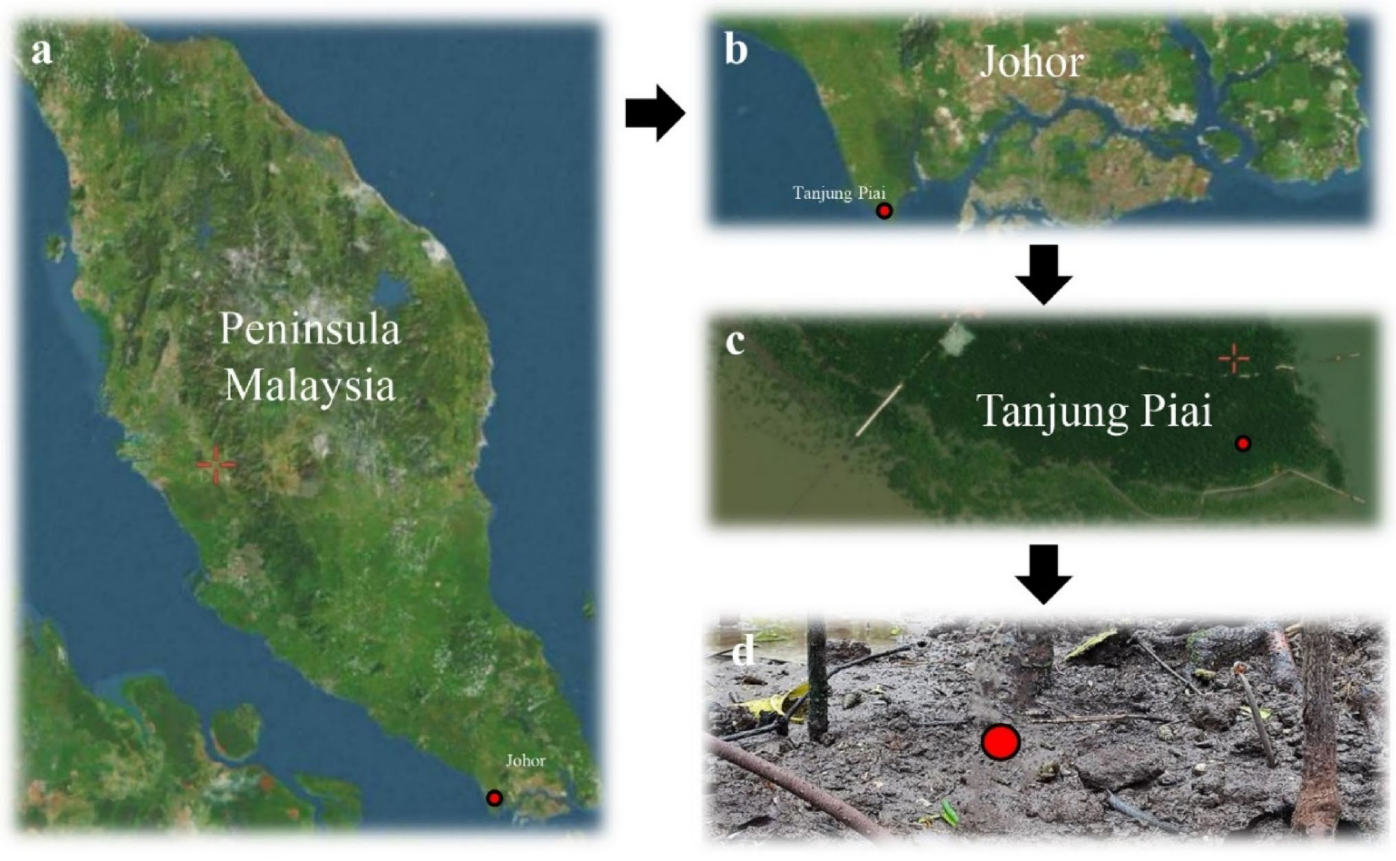
Satellite map of Malaysia showing the location of sampling at Tanjung Piai National Park. The collection site is indicated by red circles. **a** Satellite image of Peninsula Malaysia, **b** satellite image of Johor, **c** satellite image of Tanjung Piai, **d** Tanjung Piai mangrove forests sample collection site

Soil samples were collected from the Tanjung Piai National Park, Malaysia (1°16′01.6″N, 103°30′27.6″E), following the procedure described by Priya et al. (2018). The sampling site was located within a coastal wetland area (Fig. 1). Nine individual soil cores were collected from a depth of 10 cm within a 1 m^2^ plot and homogenized into one composite sample. The sampling was conducted in October 2022. The determination of physiochemical properties of soil samples was outsourced to Allied Chemists, Malaysia. Soil physiochemical parameters were measured for pH (7.4 ± 0.1), salinity (1.5% ± 0.1), and total carbon and nitrogen (C: 3.7%, N: 0.1%) The composite sample was transported to the laboratory on ice and stored at -80 °C until further process.

### DNA extraction and shotgun metagenome sequencing

Total DNA was extracted from the composite sample using the DNeasy®PowerSoil® Pro Kit (QIAGEN, Germany), following the manufacturer’s instruction. Approximately 250 mg of soil was homogenized using TissueLyser II (QIAGEN, Germany) at 25 Hz for three cycles. The quality of the extracted DNA was assessed by 1% (w/v) agarose gel electrophoresis, and DNA concentration was measured using a NanoDrop 2000 spectrophotometer (ThermoScientific, Wilmington, DE, USA). Sequencing was performed by NovogeneAIT (Singapore) using the Illumina NovaSeq 6000 platform (paired end 150 bp). The sequencing data was deposited in the NCBI Sequence Read Archive (SRA) under BioProject accession number PRJNA939948.

### Metagenomics data analysis and selection of *BGL3_GH1*

The paired-end reads in FASTQ format were quality-filtered and adapter-trimmed using Trimmomatic V0.32, retaining reads with a minimum Phred score of 20 and length ≥ 50 bp (Bolger et al. 2014). High-quality reads were assembled de novo using MEGAHIT v1.2.9 with a minimum k-mer size of 21 (Li et al. 2015). To reduce artefacts associated with highly fragmented contigs, only assembled contigs ≥ 1000 bp were retained for downstream analysis. Open reading frames (ORFs) were predicted from these filtered contigs using Prodigal v2.6.3 in metagenome mode (Hyatt et al. 2010; Rho et al. 2010). Functional annotation of ORFs was performed using eggNOG-mapper v2.1.10 and GhostKOALA with default parameters, referencing the eggNOG and KEGG databases, respectively (Cantalapiedra et al. 2021; Kanehisa et al. 2016). Carbohydrate-active enzymes (CAZymes) were identified using run_dbcan v3.0.6 (Zhang et al. 2018). Only CAZymes that were supported by two out of three methods (HMMER v3.3.2, eCAMI, or DIAMOND v2.1.6) were retained for downstream analysis. Candidate sequences were further validated against the NCBI non-redundant, RCSB Protein Data Bank (PDB), and UniProt reviewed protein databases using Diamond v2.1.6 (Buchfink et al. 2015). Protein domains of selected CAZymes were analysed using InterProScan v5.61–93.0 (Jones et al. 2014). Taxonomy of predicted CAZymes was performed using DIAMOND BLASTP against the NCBI nr database with criteria of ≥ 50% sequence identity and ≥ 50% query coverage. Based on the annotation results, a β-glucosidase gene (*BGL3_GH1*) was selected from the metagenomic data for subsequent functional characterisation and sugarcane bagasse saccharification. Domain architecture was examined using Pfam database (Bateman et al. 2004). Phylogenetic analysis was conducted by aligning BGL3_GH1 with representative GH 1 β-glucosidase retrieved from UniProt database and restricted to reviewed entries. Phylogenetic tree construction was performed using the Neighbor-Joining method with 1,000 bootstrap replicates via MEGA 11 (Tamura et al. 2021). The conserved catalytic and substrate-binding residues of recombinant BGL3_GH1 and characterized GH1 β-glucosidase were identified by performing multiple sequence alignment in Jalview 2.11.5.1 using the MUSCLE algorithm with default parameters (Waterhouse et al. 2009). The nucleotide and protein sequence data of recombinant BGL3_GH1 were deposited in the NCBI database under accession number PX852031.

### Production of the recombinant BGL3_GH1 protein

The *BGL3_GH1* gene was synthesized and codon-optimized for expression in *Escherichia coli* by Integrated DNA Technologies (IDT, Singapore). The synthesized gene and expression vector *p*ET-28a (+) were digested with EcoRI and XhoI restriction enzyme, followed by ligation using T4 DNA ligase. The recombinant plasmid was transformed into chemically competent *E*. *coli* BL21 (DE3) cells and selected on Luria–Bertani (LB) agar plates containing 50 µg mL^−1^ kanamycin. Positive transformant were cultured, harvested, and resuspended in 50 mM potassium phosphate buffer (pH 7.0). Cell lysis was carried out by sonication on ice, and the lysate was centrifuged at 4 ℃, 10,000 × g for 20 min. The supernatant was filtered through a 0.45 µm filter and loaded onto a pre-equilibrated Protino Ni–NTA columns (Macherey–Nagel). The bound protein was eluted using a linear imidazole buffer gradient (10 mM to 250 mM). The eluted recombinant BGL3_GH1 protein was dialyzed against 50 mM potassium phosphate buffer (pH 7.0). Protein purity and integrity were assessed by 12% SDS-PAGE. The protein concentration was measured using Pierce™ BCA Protein Assay Kits (Thermo Scientific), according to the manufacturer’s instructions (Liu et al. 2023).

### Determination of β-glucosidase activity

Β-glucosidase activity was assayed using *p*-nitrophenyl-β-D-glucopyranoside as substrate (*p*NPG; Sigma), following the procedure described by Jeilu et al. (2024), with slight modification. The reaction mixture consisted of 800 µL of 5 mM *p*NPG prepared in 50 mM potassium phosphate buffer (pH 7.0) and 200 µL purified enzyme. The mixture was incubated at 50 ℃ for 10 min, after which the reaction was terminated by addition of 100 µL of 1 M sodium carbonate. The release of *p*-nitrophenol (*p*NP) was quantified by measuring absorbance at 405 nm. One unit (U) of β-glucosidase activity was defined as the amount of enzyme required to release 1 µmol of *p*NP per minute under the assay condition.

### Enzymatic characterisation of recombinant BGL3_GH1

The enzymatic characterization assays were performed using BGL3_GH1 at a final protein concentration of 0.083 mg ml^−1^. The optimum pH of the enzyme was examined in different buffers: sodium acetate (pH 4.0–5.0); phosphate buffer (pH 6.0–7.0); Tris–HCl (pH 8.0–9.0); and Glycine–NaOH (pH 10.0–11.0). For pH stability assays, the enzyme was pre-incubated in the same buffer system without substrate for 240 min at room temperature, with aliquots collected every 30 min. Residual activity was quantified to a control sample without incubation.

The optimum temperature was evaluated by assaying the enzyme at temperature ranging from 5 ℃ to 80 ℃ in 5 ℃ increments. Thermostability assays were determined by pre-incubating the enzyme at temperature between 25 ℃ to 60 ℃ (5 ℃ interval) for 240 min. Aliquots were sampled every 30 min, and residual activity was measured to a control sample kept without heat treatment. To evaluate the protective effect of substrate, the enzyme was pre-incubated with *p*NPG at the same concentration as in the standard activity assay at 60 ℃ for 240 min. The residual activity was measured every 30 min.

The effect of glucose on enzyme activity was assessed by measuring in the presence of glucose concentrations from 0 to 3000 mM. Background control containing the substrate and glucose without enzyme were included to correct for any absorbance interference. Reaction rates were measured during the initial assay period at all glucose concentrations to confirm linearity. Relative activity was calculated by comparing to control sample without glucose. Salt tolerance was determined by assaying enzyme with NaCl concentration from 1 to 10% (w/v). The relative activity was measured by comparing to control without salt.

The influence of various metal ions on enzyme activity was evaluated by including KCl, NiSO_4_, MnCl_2_, CoCl_2_, AlCl_3_, FeCl_2_, ZnCl_2_, MgCl_2_, CaCl_2_, SrCl_2_, and CuCl_2_ (final concentration of 5 mM) in the reaction mixture. The relative activity was calculated by comparing to a control without metal ions.

The effects of surfactants and inhibitors were studied by adding 5% (v/v) of chemical additives to the reaction mixture. The chemical additives include dimethyl sulfoxide (DMSO), glycerol, Tween-20, Tween-40, Tween-80, Triton X-100, Ethylenediaminetetraacetic acid (EDTA), Sodium dodecyl sulfate (SDS), and urea. The relative activity was determined by comparison with a control without chemical additives.

The kinetic parameters of the enzyme were determined by measuring enzyme activity at varying *p*NPG concentrations from 0.1 mM to 20 mM. Data were fitted to the Michaelis–Menten equation using non-linear regression in GraphPad Prism with 95% confidence intervals. Since enzyme kinetics were determined on recombinant BGL3_GH1 that was purified by a single-step purification method, maximum reaction velocity (*V*_*max*_) and turnover number (*k*_*cat*_) were reported as apparent values to account for minor contaminants.

### Two-step degradation of sugarcane bagasse

**Fig. 2:**
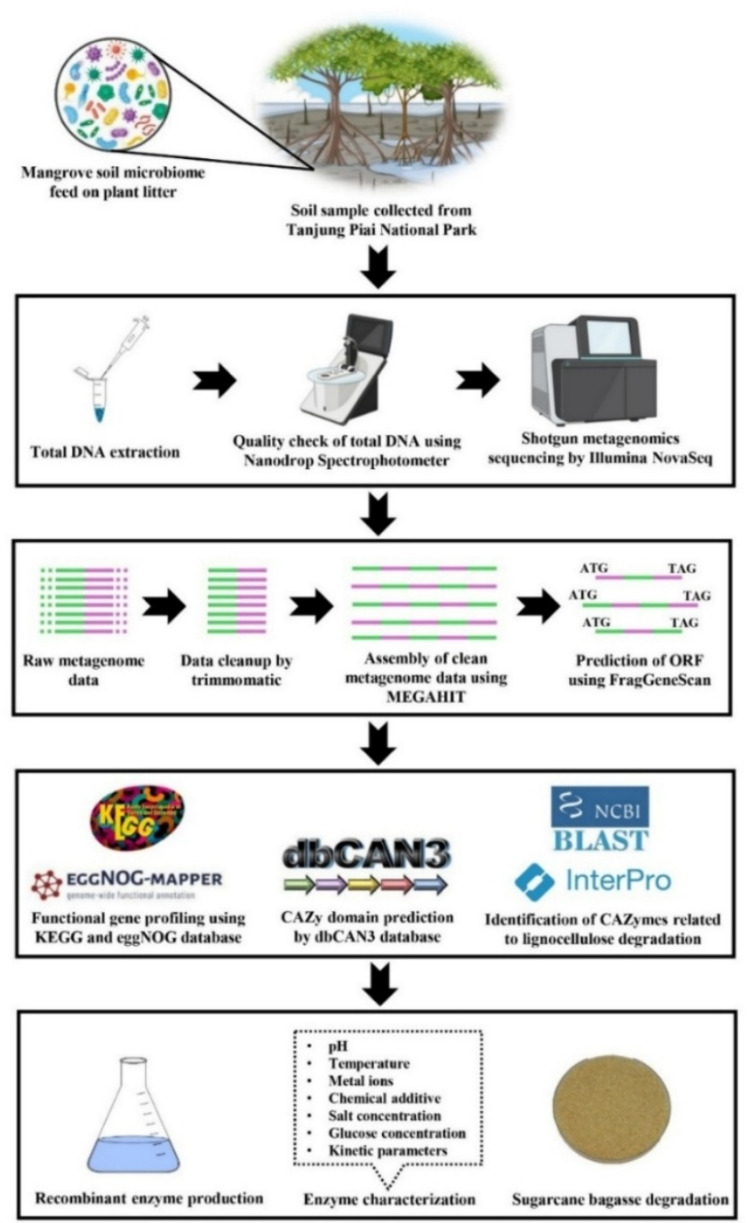
Overall workflow of mangrove lignocellulose degrading gene analysis and functional characterisation of GH 1 β-glucosidase (BGL3_GH1)

Sugarcane bagasse was collected from a local sugarcane juice vendor. The bagasse was washed thoroughly to remove impurities, and oven-dried at 60 ℃ to remove moisture. The dried sugarcane bagasse was ground and sieved to obtain a particle size of 1 mm. For enzymatic hydrolysis, 2.5% (w/v) of the processed biomass was suspended in 50 mM potassium phosphate buffer (pH 7.0). Enzymatic hydrolysis was conducted in commercial cellulase only, recombinant BGL3_GH1 only, and combined commercial cellulase and recombinant BGL3_GH1. All reactions were performed at 60 ℃ with shaking at 150 rpm for a total incubation time of 4 h. For the commercial cellulase only treatment, the biomass suspension was incubated with a commercial cellulase preparation, Cellic® CTec (Novozymes, Denmark) at an enzyme loading of 10 FPU g^−1^ dry biomass for 4 h. The recombinant BGL3_GH1-only treatment was incubated with recombinant BGL3_GH1 at an enzyme loading of 10 U g⁻^1^ dry biomass for 4 h. For the combined enzyme treatment, the biomass suspension was first hydrolyzed with commercial cellulase at 10 FPU g^−1^ dry biomass for 2 h. The reaction mixture was then centrifuged at 10,000 × g for 5 min, and the supernatant was supplemented with recombinant BGL3_GH1 at 10 U g^−1^ dry biomass. The reaction mixture was then incubated for an additional 2 h under the same condition. Control reaction without enzyme were included to account for non-enzymatic sugar release. All experiments were performed in triplicate. The concentration of reducing sugar in the final hydrolysate was determined using the 3,5-dinitrosalicylic acid (DNS) method as described by Gusakov et al. (2011). The overall workflow was illustrated in Fig. 2.

### Statistical analysis

All experimental assays were performed using three independent biological replicates, with each biological replicates measured in technical triplicate. The data are presented as means ± standard deviation. Statistical significance among different experimental condition (pH, temperature, NaCl concentration, glucose concentration, and enzymatic treatments) was evaluated using one-way ANOVA with Tukey’s post hoc test. Differences were considered statistically significant at* p* < 0.05.

## Results

### Overview of metagenomics data

A total of 20.9 GB of raw shotgun metagenomic sequencing data in paired-end FASTQ format was obtained. The dataset comprised 67,646,094 pair-end reads. After adapter removal using Trimmomatic, 99.96% of the reads remained as clean data, with a GC content was 54.71%. Assembly of the clean reads using MEGAHIT produced 2,544,877 contigs with an N50 of 542 base pairs (Table 1). To improve reliability of downstream analysis, only contigs ≥ 1,000 bp were retained for ORF prediction and downstream analysis. The contigs length distribution was illustrated in Fig. S1. The filtering step resulted in 173,623 contigs with a total length of 272.9 Mb, and an average contig length of 1,571 base pairs. A total of 367,595 open reading frames (ORFs) were predicted from the filtered contigs. The ORF length distribution demonstrated that approximately 25% of the predicted genes exceed 300 amino acids (Fig. S2). This indicates the presence of long ORFs for functional annotation. Thus, the ORFs were used in subsequent analysis.

**Table 1:** Overview of metagenomics data

Parameters	TP mangrove soil sample
Total number of paired-end reads	67,646,094
Total number of clean paired-end reads	67,622,916
Q30 bases of clean reads	93.66%
Q20 bases of clean reads	97.81%
G + C content of clean reads	54.71%
Total number of contigs length ≥ 200 bp	2,544,877
Total assembly size (bp)	1,392,726,341
N50 (bp)	542
Total number of ORFs predicted from contigs ≥ 1,000 bp	367,595
Total number of functional genes predicted from contigs ≥ 1,000 bp	183,487
BioProject ID	PRJNA939948
BioSample ID	SAMN33549506
SRA accession number	SRR23652001

### Functional analysis of metagenomics dataset

**Fig. 3:**
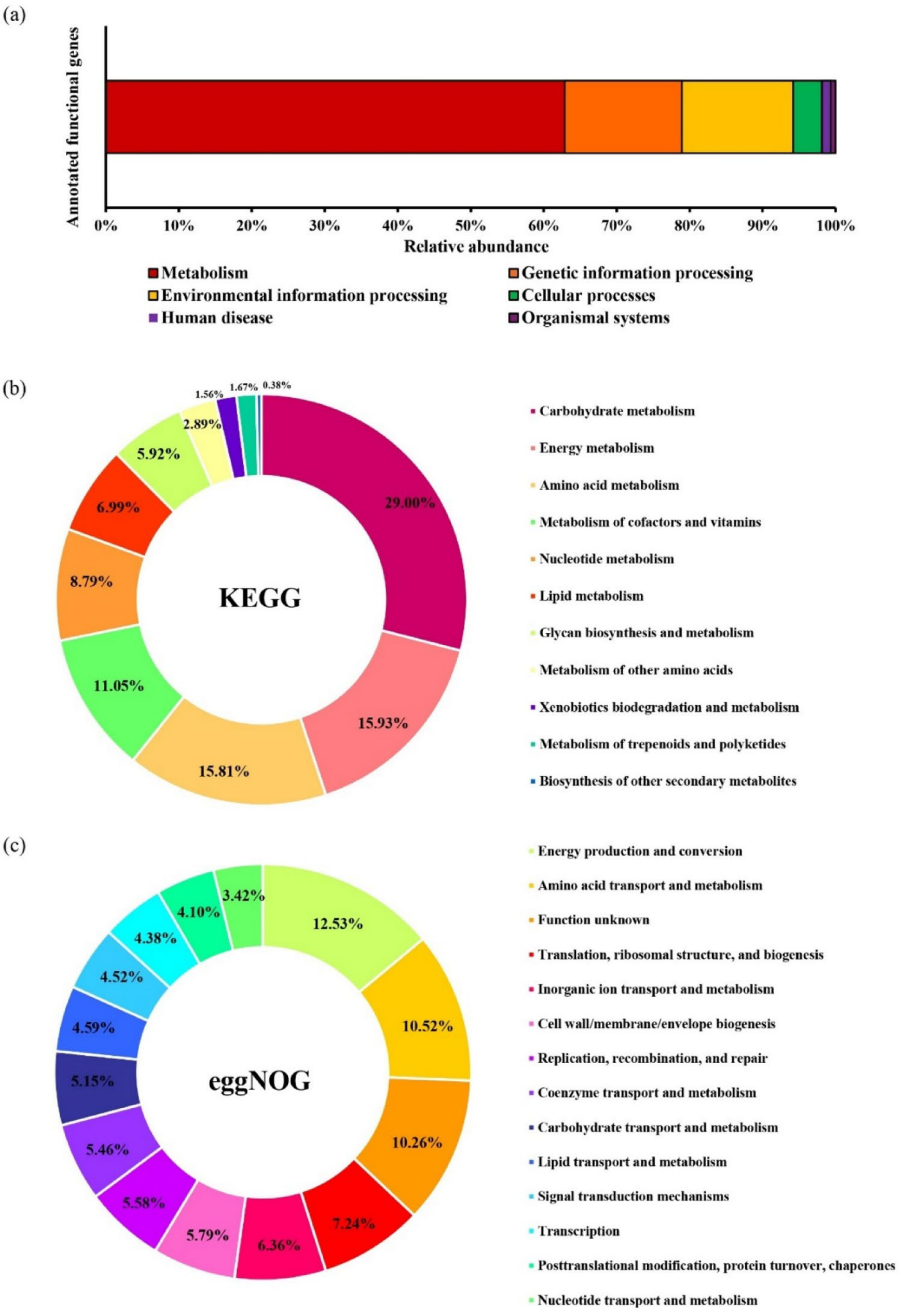
Functional gene profile of TP mangrove soil microbiome based on ORFs predicted from contigs ≥ 1,000 bp. **a** Relative abundance of annotated functional genes across major KEGG pathways. **b** Distribution of annotated genes among metabolic sub-pathways within the KEGG metabolism category. **c** Functional classification of metagenomic genes based on eggNOG-mapper annotation showing relative abundances of genes assigned to different functional categories

The functional gene profile of the bacterial community was determined by annotating predicted open-reading frames (ORFs) from contigs ≥ 1,000 bp against the KEGG and eggNOG databases. KEGG-based analysis revealed that most functional genes were assigned to the Metabolism category, accounting for 62.89% of the total annotated genes. This was followed by genetic information processing (16.04%) and environmental information processing (15.25%). The cellular processes category comprised 3.94% of the annotated genes, while human disease (1.23%) and organismal systems categories (0.64%) were the least represented (Fig. 3a). Further examination within the metabolism category revealed a diverse functional potential among the bacterial communities. Carbohydrate metabolism was the most abundant pathway (29.00%), followed by energy metabolism (15.93%) and amino acid metabolism (15.81%), respectively. The least represented metabolism pathway included xenobiotics biodegradation and metabolism (1.67%), metabolism of terpenoids and polyketides (1.56%), and biosynthesis of other secondary metabolites (0.38%) (Fig. 3b). Functional annotation using eggNOG exhibited a comparable distribution to the KEGG results (Fig. 3c). Functional categories such as energy production and conversion, amino acid transport and conversion, and carbohydrates transport and metabolism were among the most represented, comprising of 12.53%, 10.52%, and 5.15%, respectively.

### Mining of carbohydrate degrading genes from metadata

**Fig. 4:**
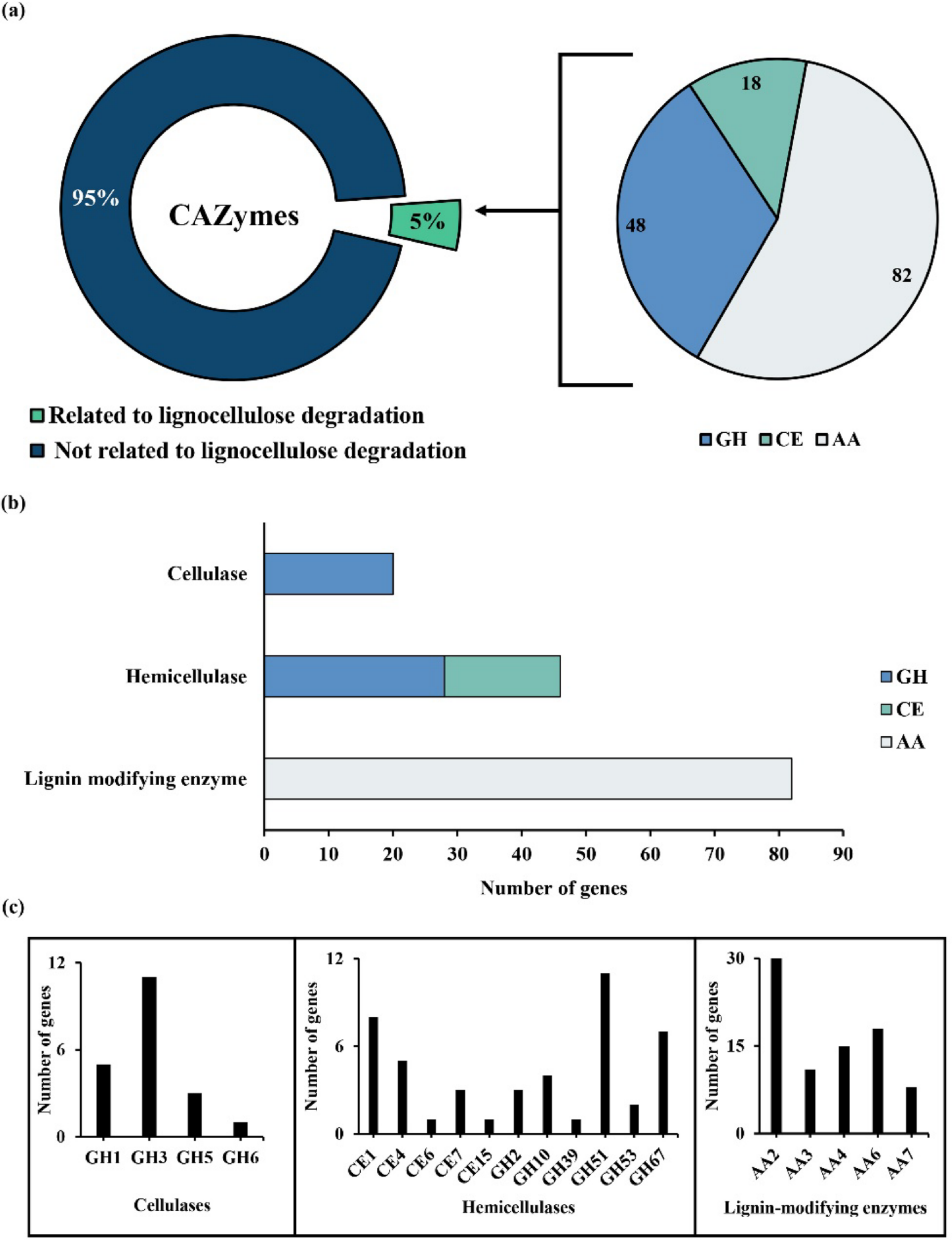
CAZyme domains annotated in the metagenomic dataset with contigs ≥ 1,000 bp. *GH* Glycosyl hydrolase, * AA* Auxiliary activity,* CE* Carbohydrate esterase. **a** Lignocellulose-related CAZyme domains identified based on dbCAN and InterProScan annotation; **b** Functional classification of lignocellulolytic CAZymes into cellulase, hemicellulase, and lignin-modifying enzyme; **c** CAZy family-level composition of cellulase, hemicellulase, and lignin-modifying enzyme in contribution to specific GH, CE, and AA families to lignocellulose degradation

A total of 3,035 CAZymes were identified from the filtered contigs which distributed among all six major classes. Among these CAZy families, GHs were highly represented, accounting for 47.31% of the annotated CAZymes, followed by the GTs at 38.10%. The CBM, CE, and AA families constituted 7.63%, 3.62%, and 2.97% respectively. PLs were the least abundant, comprising with only 0.37%. Further investigation identified 148 CAZymes putatively involved in lignocellulose degradation. These lignocellulolytic genes were distributed among GH (48 ORFs), CE (18 ORFs), and AA (82 ORFs) families (Fig. 4a). Based on their enzymatic functions, these genes were categorized into cellulases (20 genes), hemicellulases (46 genes), lignin-modifying enzymes (82 genes) (Fig. 4b). At the CAZy family level, cellulolytic genes were mainly represented by GH 1, 3, 5, and 6. The hemicellulases were determined with CE (CE 1, 4, 6, 7, and 15) and GH (GH 2, 10, 39, 51, 53, and 67) families. Lignin modification was primarily mediated by AA 2, 3, 4, 6, and 7 (Fig. 4c).

### Classification of lignocellulose degrading genes

Analysis of lignocellulolytic genes revealed 20 CAZy families from the GH, CE, and AA classes associated with lignocellulose degradation. The cellulases group included of β-glucosidase (GH 1 and 3), endoglucanase (GH 5), and cellobiohydrolase (GH 6). These cellulases were encoded by bacterial genera such as *Aurantiacibacter*, *Bacteroides*, *Paraglaciecola*, and *Pseudolabrys* (Table 2).

**Table 2:** Taxonomic affiliation of lignocellulose degrading CAZymes identified from contigs ≥ 1,000 bp based on DIAMOND BLASTP assignment (≥ 50% sequence identity and ≥ 50% query coverage)

Enzymes	CAZy families	Detected bacteria communities
*Cellulases*		
β-glucosidase (EC 3.2.1.21)	GH 1, 3	*Anaerolineales*, *Acidimicrobiia*, *Aurantiacibacter*, *Bacteroides*, *Gemmatimonadales*, *Kiloniellaceae*, *Pseudolabrys*, *Streptosporangiales*
Endoglucanase (EC 3.2.1.4)	GH 5	*Anaerolineae*, *Deltaproteobacteria*, *Paraglaciecola*
Cellobiohydrolase (EC 3.2.1.91)	GH 6	*Streptosporangiales*
*Hemicellulases*		
Acetyl xylan esterase (EC 3.1.1.72)	CE 1, 6, 7, 15	*Acidobacteriota*, *Anaerolineae*, *Aquisphaera*, *Gemmatimonadales*, *Mycobacterium*, *Mycolicibacterium*, *Paenibacillus*, *Planctomycetota*, *Prolixibacteraceae*, *Verrucomicrobiales*
Polysaccharide deacetylase (EC 3.1.1.58)	CE 4	*Hyphomicrobiales*, *Hyphomicrobiaceae*, *Pseudolabrys*
α-glucuronidase (EC 3.2.1.39)	GH 2, 67	*Acidobacteriota*, *Anaerolinea*, *Sphingomonas*,* Thermoflexales*
α-arabinofuranosidase (EC 3.2.1.55)	GH 51	*Acidobacteriota*, *Ardenticatenaceae*, *Bacteroidales*, *Bacteroides*, *Chloroflexi*, *Deltaproteobacteria*, *Luteitalea*
Arabinogalactan endo-1,4-β-galactanase (EC 3.2.1.89)	GH 53	*Anaerolineaceae*, *Trinickia*
β-xylanase (EC 3.2.1.8)	GH 10	*Anaerolineae*, *Deltaproteobacteria*, *Pyrinomonadaceae*
β-xylosidase (EC 3.2.1.37)	GH 39	*Acidobacteriota*
*Lignin-modifying enzyme*		
Catalase-peroxidase (EC 1.11.1.21)	AA 2	*Anaerolinea*, *Acidithiobacillales*, *Acidimicrobiales*, *Bauldia*, *Deltaproteobacteria*, *Desulfobacterales*, *Desulfofustis*, *Gammaproteobacteria*, *Methyloligella*, *Phycisphaerae*, *Thiohalobacter*, *Solirubrobacterales*
GMC family oxidoreductase (EC 1.1.99.B3)	AA 3	*Betaproteobacteria*, *Filomicrobium*, *Gammaproteobacteria*, *Hyphomicrobiales*, *Hyphomicrobiaceae*, *Mycobacterium*, *Rhodocyclaceae*
FAD-binding oxidoreductase (EC 1.1.5.9)	AA 4, 7	*Alphaproteobacteria*, *Anaerolineae*, *Chloroflexi*, *Deltaproteobacteria*, *Desulfobacterales*, *Gammaproteobacteria*, *Gemmatimonadia*, *Hyphomicrobiales*, *Hyphomicrobiaceae*, *Methyloceanibacter*, *Mycobacterium*, *Paraburkholderia*, *Plantomycetota*,* Pseudolabrys*
NADPH quinone oxidoreductase (EC 1.6.5.5)	AA 6	*Burkholderiaceae*, *Deltaproteobacteria*, *Desulfobacterales*, *Desulfobacca*, *Geminicoccaceae*, *Methyloceanibacter*, *Nitrospira*, *Nitrospirota*

*GH* Glycosyl hydrolase, *AA* Auxiliary activity, *CE* Carbohydrate esterase

A total of 11 CAZy families were identified with of hemicellulolytic function, including acetyl xylan esterase (CE 1, 6, 7, and 15), polysaccharide deacetylase (CE 4), α-glucuronidase (GH 2, 67), α-arabinofuranosidase (GH 51), arabinogalactan endo-1,4,-β-galactanase (GH 53), β-xylanase (GH 10), and β-xylosidase (GH 39). These genes were found in diverse bacteria genera such as *Aquisphaera, Bacteroides*, *Luteitalea*, *Mycobacterium*, *Mycolicibacterium*, *Paenibacillus*, *Pseudolabrys*, *Sphingomonas*, and *Trinickia* (Table 2).

Lignin-modifying enzymes were represented by catalase-peroxidase (AA 2), GMC family oxidoreductase (AA 3), FAD-binding oxidoreductase (AA 4 and 7), and NADPH quinone oxidoreductase (AA 6). These enzymes were encoded by bacteria genera such as *Bauldia*, *Desulfobacca*, *Desulfofustis*, *Filomicrobium*, *Methyloceanibacter*, *Methyloligella*, *Mycobacterium*, *Nitrospira*, *Paraburkholderia*, *Pseudolabrys*, and *Thiohalobacter* (Table 2). These findings highlight the enzymatic profile of bacterial communities for lignocellulose degradation.

**Fig. 5:**
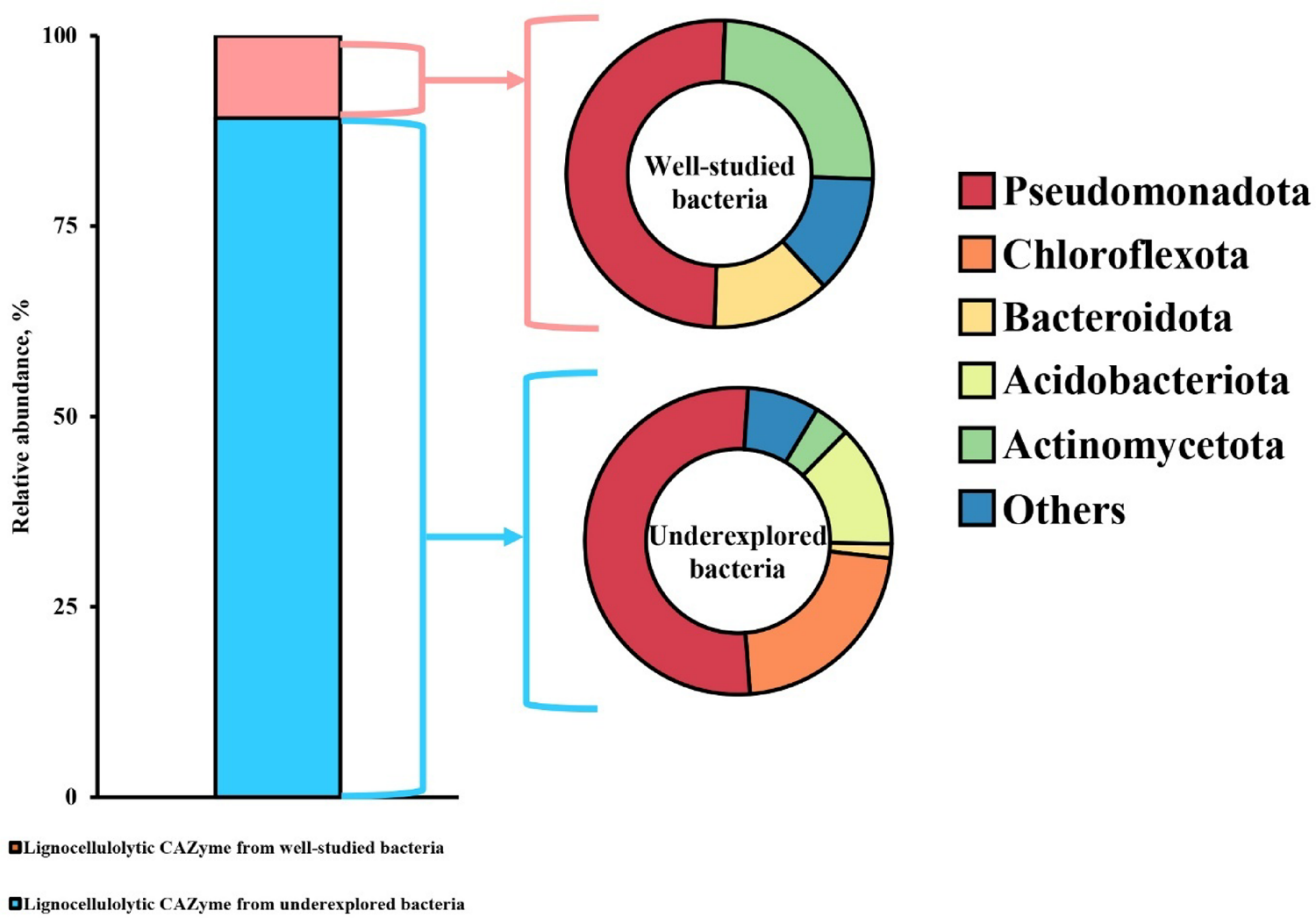
Taxonomic resolution of lignocellulolytic CAZymes identified from contigs ≥ 1,000 bp. The bar chart shows the relative abundance of lignocellulolytic genes in both well-studied and underexplored bacteria lineages based on DIAMOND BLASTP thresholds (≥ 50% sequence identity and ≥ 50% query coverage); The donut charts depict the taxonomy composition on phyla level of well-studied bacteria (top) and underexplored bacteria (bottom) taxa annotated from the lignocellulolytic CAZymes

In this study, we defined the underexplored bacteria as (i) those that are unable to be assigned into genus level, (ii) genera consisting of limited species (< 5 species) listed in the LPSN database, or (iii) limited studies on protein functional characterization. Among the 148 CAZymes, 89.19% (132 genes) were categorized as underexplored bacteria, while the reaming 10.81% (16 genes) were identified from bacteria lineages that have been extensively studied (Fig. 5). Within the well-studied category, most genes were affiliated with the phylum Pseudomonadota (50.00%, 8 genes), followed by Actinomycetota (25.00%, 4 genes) and Bacteroidota (12.5%, 2 genes). In the underexplored category, Pseudomonadota was also the prominent phylum, accounting for 52.27% (69 genes), followed by Chloroflexota (22.00%, 29 genes), Acidobacteriota (7.58%, 10 genes), Actinomycetota (3.79%, 5 genes), and Bacteroidota (1.52%, 2 genes). The distribution of lignocellulolytic genes between well-studied and underexplored bacteria were illustrated at Fig. 5.

### Synergy action of bacterial communities in lignocellulose degradation

**Fig. 6:**
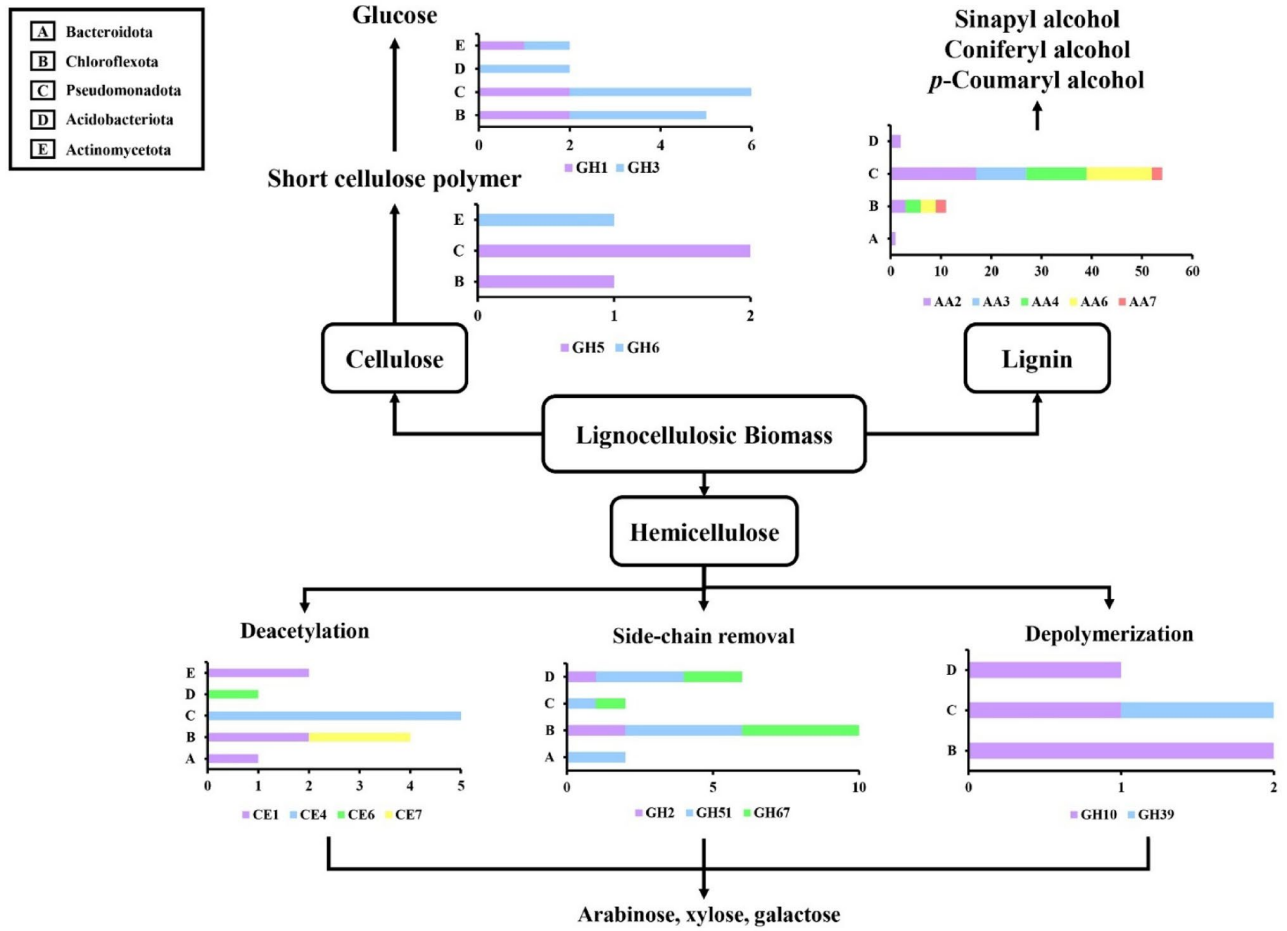
Proposed synergistic model for lignocellulose degradation by mangrove bacterial phyla. *GH* Glycosyl hydrolase, *AA* Auxiliary activity, *CE* Carbohydrate esterase. The bar chart indicates the distribution of CAZymes domains across different phyla involved in the degradation of specific lignocellulose polymers. y-axis: Bacterial phylum; x-axis: gene count

Further analysis revealed that lignocellulolytic enzymes from diverse bacterial communities act synergistically to degrade lignocellulose. The enzymatic degradation of cellulose, hemicellulose, and lignin by mangrove bacterial communities were illustrated in Fig. 6. Five different bacteria phyla (Acidobacteriota, Actinomycetota, Bacteroidota, Chloroflextota, and Pseudomonadota) were found to participate in the degradation process. Pseudomonadota dominated cellulose degradation, followed by Chloroflexota, Actinomycetota, and Acidobacteriota. Lignocellulolytic genes associated with Bacteriodota phyla were not detected in the cellulose degradation pathway. β-glucosidase (GH 3) were the dominant cellulase across five phyla. Notably, the cellobiohydrolase (GH 6) families were unique to Actinomycetota. The combined action of these cellulases can degrade cellulose into simple sugar, glucose (Fig. 6). Lignin degradation was primarily driven by Pseudomonadota phylum (54 genes), followed by Chloroflexota (11 genes), Actinomycetota (2 genes), and Bacteroidota (1 gene), while Actinomycetota was not assigned with any AA families. Most lignin-degrading enzymes were discovered in AA 2 (catalase-peroxidase), followed by others auxiliary enzymes from AA 3, 4, 6, and 7. These lignin-modifying enzymes were able to oxidize lignin into monolignols such as sinapyl alcohol, coniferyl alcohol, and *p-*Coumaryl alcohol (Fig. 6).

Furthermore, CAZymes linked to hemicellulose degradation were detected that involved in deacetylation, side-chain removal, and backbone depolymerization. Majority of the hemicellulase genes were related in removing side-chain and acetyl group from the hemicellulose backbone. Deacetylation was primarily mediated by CE 1, 4,6, and 7, with CE 4 showing the highest contribution. The degradation of carbohydrate ester was predominant by CE 4 from the Pseudomonadota phylum (5 genes) and followed by Chloroflexota (4 genes). Notably, five bacterial phyla were detected in the deacetylation of hemicellulose. The side-chain removal was driven by GH 2, 51, and 67, which the predominant phyla was Chloroflexota (10 genes) and Acidobacteriota (6 genes). Hemicellulases in depolymerization of hemicellulose backbone were associated with Chloroflexota, Pseudomonadota, and Acidobacteriota. The primary enzymes in hemicellulose depolymerization were GH 10 and 39. The sequential degradation of hemicellulose by these hemicellulases will produce monosaccharides such as arabinose, xylose, and galactose. These results revealed the functional specialization and cooperative roles of mangrove bacterial communities in lignocellulose degradation.

### Production and characterisation of recombinant BGL3_GH1

Among the 148 lignocellulolytic CAZymes identified, a β-glucosidase gene belonging to the GH 1 family (designated *BGL3_GH1*) was selected for experimental characterization. The gene was selected due to (i) the gene was determined from the underexplored bacteria group (ii) industrial relevance as β-glucosidase plays an important role in cellulose degradation by catalyzing the terminal hydrolysis of cellooligosaccharides into glucose. Thus, *BGL3_GH1* was chosen as the representative enzyme to validate the functional potential derived from the metagenomic analysis. The selected gene was predicted with the presence of a complete GH 1 catalytic domain based on InterProScan analysis. Recombinant BGL3_GH1 protein was successfully expressed and purified using a single-step affinity chromatography approach. SDS-PAGE analysis on partially purified recombinant BGL3_GH1 protein revealed a dominant band at approximately 50 kDa which corresponds to the expected molecular mass of recombinant BGL3_GH1 (Fig. S3). This indicates that the recombinant BGL3_GH1 was suitable for subsequent biochemical characterization. In silico analysis further confirmed the functional annotation of recombinant BGL3_GH1, including the presence of a complete GH 1 catalytic domain (Fig. S4a), phylogenetic clustering with characterized GH 1 β-glucosidase (Fig. S4b), and identification of key catalytic and substrate-binding residues of GH 1 enzymes (Fig. S4c).

**Fig. 7:**
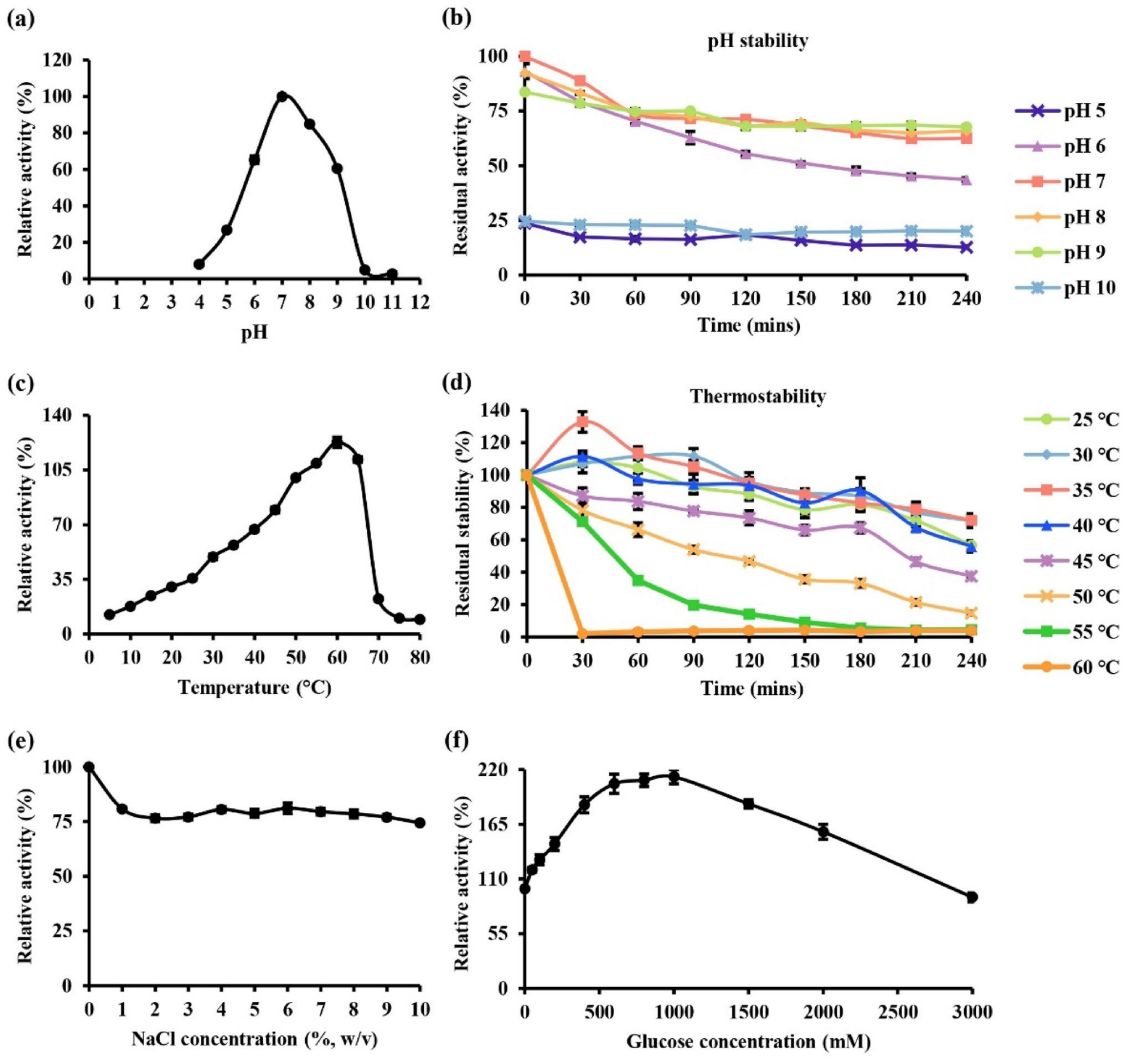
Functional characterisation of recombinant BGL3_GH1 showing **a** optimal pH; **b** pH stability over a 240-min incubation period; **c** optimal temperature; **d** thermostability of over a 240-min incubation period; **e** effect of NaCl concentrations; **f** effect of glucose concentration. Data were presented as mean ± standard deviation. Statistical significance among conditions was assessed where applicable using one-way ANOVA (*p* < 0.05)

Recombinant BGL3_GH1 demonstrated the highest activity at pH 7. The relative activity of the enzyme decreased at pH values below 6 and above 9, falling to less than 30% of the maximum activity (Fig. 7a). It remained most stable between pH 7 and 9, retaining around 70% of its residual activity. However, at pH 5 and 10, the enzyme activity decreases sharply to approximately 25% (Fig. 7b). The differences in activity across pH conditions were statistically significant (*p* < 0.05). Temperature profiling revealed that high enzymatic activity between 50 ℃ and 65 ℃ (≥ 90%), with maximal activity at 60 ℃. At temperature above 65 ℃, the relative activity declined sharply to below 25% (*p* < 0.05) (Fig. 7c). Thermostability analysis showed that recombinant BGL3_GH1 was most stable at 30 ℃ and 35 ℃, retaining more than 70% residual activity. However, the residual activity decreased when temperature increased, reaching approximately 4% at 60 ℃ (Fig. 7d). This suggests that elevated temperatures favor catalytic activity but compromise long-term enzyme stability.

Maximal relative activity was observed in the absence of salt. Notably, in the presence of NaCl concentration ranging from 1 to 10% (w/v), recombinant BGL3_GH1 retained over 75% of its maximal activity (Fig. 7e). Recombinant BGL3_GH1 exhibited increased enzymatic activity in the presence of glucose concentrations up to 2,000 mM, showing a 1.57-fold to 2.12-fold increase compared to glucose-free control (*p* < 0.05). The enzyme also tolerated glucose concentrations up to 3,000 mM, retaining approximately 90% of its maximal activity (Fig. 7f). No background absorbance was detected in enzyme-free controls, and reaction rates remained linear for all glucose concentrations tested.

The activity of recombinant BGL3_GH1 was evaluated in the presence of various metal ions and chemical additives (Table 3). Potassium (K^+^) and magnesium (Mg^2+^) have minimal effects on enzyme activity, with more than 90% relative activity retained and no significant different compared to the control. In contrast, recombinant BGL3_GH1 activity was substantially inhibited by strontium (Sr^2+^), calcium (Ca^2+^), zinc (Zn^2+^), copper (Cu^2+^), iron (Fe^2+^), nickel (Ni^2+^), and aluminum (Al^3+^), with relative activity reduced to below 30% (*p* < 0.05). Among the chemical additives tested, glycerol and the non-ionic surfactants such as Tween-20, Tween-40, Tween-80, and Triton X-100 enhanced recombinant BGL3_GH1 activity, resulting in approximately 1.6-fold to 2.2-fold increases compared to the control (*p* < 0.05). In contrast, the chelating agent, EDTA and anionic detergent, SDS significantly reduced the enzyme activity to below 20% (*p* < 0.05). The sensitivity of recombinant BGL3_GH1 to metal ions and EDTA suggests that metal-dependent interaction may influence enzyme structure or catalytic efficiency. As the enzyme preparation was obtained using a single-step affinity purification, the kinetic parameters were interpreted as apparent values. Apparent kinetic parameters were estimated by non-linear regression using *p*NPG at concentration ranging from 0.1 mM to 20 mM, with initial reaction rates pooled from three independent biological replicates (n = 60 total data points) (Fig. S5). Recombinant BGL3_GH1 exhibited an apparent maximum reaction velocity, *V*_max_ of 22.93 µmol min^−1^ mg^−1^ and an apparent Michaelis constant, *K*_m_, of 2.16 mM. The apparent turnover number (*k*_cat_) was calculated to be 276.2 s^−1^, resulting in an apparent catalytic efficiency (*k*_cat_ /* K*_m_) of 127.76 s^−1^ mM^−1^.

**Table 3:** The effects of metal ions and chemical additives on the activity of recombinant BGL3_GH1

Chemicals	Relative activity (%)
Control	100.00 ± 0.01
K^+^	99.74 ± 1.23
Mg^2+^	90.55 ± 1.26
Co^2+^	70.12 ± 0.62*
Mn^2+^	51.82 ± 3.77*
Sr^2+^	43.94 ± 3.75*
Ca^2+^	42.69 ± 0.17*
Zn^2+^	30.14 ± 0.92*
Cu^2+^	25.20 ± 1.21*
Fe^2+^	20.65 ± 4.10*
Ni^2+^	19.55 ± 2.38*
Al^3+^	12.02 ± 0.49*
Dimethyl sulfoxide	97.61 ± 8.45
Glycerol	222.86 ± 2.77*
Tween-20	177.92 ± 4.95*
Tween-40	170.10 ± 2.11*
Tween-80	142.17 ± 6.05*
Triton X-100	164.90 ± 0.67*
Ethylenediaminetetraacetic acid	18.90 ± 1.95*
Sodium dodecyl sulfate	6.13 ± 0.37*
Urea	80.07 ± 4.86*

Data are presented as mean ± standard deviation

Statistical significance relative to the control was determined using one-way ANOVA followed by Tukey’s post hoc test, *p < 0.05

### Sugarcane bagasse degradation by two-step degradation

The lignocellulolytic potential of recombinant BGL3_GH1 was evaluated using sugarcane bagasse as the substrate in a two-step hydrolysis process, consisting of an initial treatment by commercial cellulases followed by supplementation with BGL3_GH1. Recombinant BGL3_GH1 alone produced a saccharification yield of 0.0354 ± 0.0041 g g^−1^, while commercial cellulase alone resulted in a significantly higher saccharification yield of 0.0534 ± 0.0011 g g^−1^ (*p* < 0.05). The combined application of commercial cellulase and recombinant BGL3_GH1 produced the highest saccharification yield of 0.0674 ± 0.0022 g g^−1^, which was significantly greater than the commercial cellulase alone (*p* < 0.05) (Table 4). These results indicate that supplementation with BGL3_GH1 enhances cellulase-mediated saccharification efficiency, supporting its potential application in biomass hydrolysis.

**Table 4:** Saccharification yield from sugarcane bagasse degradation under different degradation condition by commercial cellulase and recombinant BGL3_GH1 in a two-step hydrolysis process

Degradation condition	Saccharification yield (g g^−1^ biomass)
Recombinant BGL3_GH1 only	0.0354 ± 0.0041
Commercial cellulase only	0.0534 ± 0.0011*
Commercial cellulase + recombinant BGL3_GH1	0.0674 ± 0.0022**

Data are presented as mean ± standard deviation

Asterisks * and ** indicate significant differences compared with recombinant BGL3_GH1 alone and commercial cellulase alone, respectively, using one-way ANOVA followed by Tukey’s post hoc test, *p < 0.05

## Discussion

Mangroves are intertidal forests that spread along the coastlines with periodic tidal flooding driving shifts in environmental conditions. This influences the mangrove bacterial communities and shaping both their taxonomic composition and functional capabilities (Imchen and Kumavath 2020). Previous culture-dependent methods have demonstrated that mangrove microbes possess diverse metabolic traits, including the ability to degrade lignocellulosic polymers through the production of cellulase, hemicellulase, and lignin modifying enzymes (Das et al. 2023; Lam et al. 2020; Pramono et al. 2021). However, majority of bacterial diversity and its functional potential remain inaccessible through conventional cultivation techniques (Bodor et al. 2020). In this study, a metagenomic approach was employed to investigate the functional gene repertoire and lignocellulolytic potential of mangrove soil microbiome from Tanjung Piai National Park, Johor.

Identifying the functional traits of bacterial communities in an environment is crucial for understanding ecosystem processes and predicting microbial responses to environmental changes. In this study, the functional gene profile of mangrove bacterial was determined using the KEGG, eggNOG, and CAZy databases. Functional annotation revealed that a significant number of ORFs were assigned to key metabolic pathways, particularly in carbohydrates metabolism. These functional genes primarily include housekeeping genes essential for maintaining bacterial life in the mangrove environment. This result aligns with other mangrove metagenomic studies from China and India, which also reported a high abundance of functional genes associated with carbohydrate metabolism (Ghosh et al. 2022; Priya et al. 2018; Zhao et al. 2019). The prominence of carbohydrate metabolism in the mangrove microbiome is likely due to the abundance of plant litter in the mangrove environment. Mangrove plant litters is rich in carbohydrates such as arabinose, galactose, glucose, and xylose, primarily in the form of cellulose and hemicellulose (Mujtaba et al. 2023). As a result, mangrove ecosystems favor the growth of lignocellulose degrading bacteria as a natural means for processing the abundance organic matter, in order to maintain the health of the ecosystems (Kathiresan 2019).

The carbohydrate utilization potential of bacterial communities was further assessed by annotating the predicted ORFs using the CAZy database. This provides detailed insights into the carbohydrate metabolism of the bacterial communities. A total of 3,035 CAZy genes were identified and distributed across six different CAZy families. These annotated enzymes were associated with the degradation of various carbohydrate substrates, including alginate, carrageenan, chitin, pectin, starch, and lignocellulose. The identification of these CAZy families highlights the metabolic potential of the mangrove bacterial communities in processing a diverse range of complex carbohydrate (Zhang et al. 2023). As plant litters in mangrove are mainly made up of lignocellulose components comprises of cellulose, hemicellulose, and lignin (Krishna and Mohan 2017). In this study, 148 lignocellulolytic genes encoding cellulases, hemicellulases, and lignin-modifying enzymes from various GH, CE, and AA families were determined from the metagenomic dataset. The proportion of lignocellulolytic genes in our dataset (approximately 5%) and the dominance of GH and GT families are consistent with those reported in other mangrove soil metagenome studies, which also reported CAZy profiles enriched in GH and GT families (Wang et al. 2025; Yahaya et al. 2024). Despite the total CAZy gene count in this study was relatively lower than that reported in previous mangrove metagenomes, the comparable distribution of CAZy classes suggests that the mangrove bacterial communities retain a strong functional potential for plant litter degradation. Taxonomic assignment revealed that majority of the lignocellulolytic CAZy genes originated from phylum Pseudomonadota, Chloroflexota, Bacteroidota, Acidobacteriota, and Actinomycetota. Notably, out of 148 lignocellulolytic genes, 89.19% were derived from underexplored bacteria taxa. These included genera such as *Anaerolinea, Bauldia*, *Luteitalea*, and *Pseudolabrys*. The predominance of lignocellulose degrading genes from the underexplored bacteria indicates a valuable genetic resource for further biotechnological exploration.

The lignocellulolytic gene pools identified in this study reflects a coordinated enzymatic framework for lignocellulose biomass degradation. A set of cellulases, including β-glucosidase (GH 1 and 3), endoglucanase (GH 5), and cellobiohydrolase (GH 6) were detected in this study. These enzymes are essential for the efficient degradation of cellulose as they act on the β-(1,4)-glycosidic bond in cellulose and short cellulose polymer to produce glucose molecules as the final product (Soni et al. 2018). Similar findings have been reported in other metagenomic studies, which β-glucosidase (GH 3) and endoglucanase (GH 5) families were typically detected as well (Ghose et al. 2024; Paixão et al. 2021; Parab et al. 2024; Priya et al. 2018; Zhang et al. 2023). However, cellobiohydrolase (GH 6) was poorly represented in those studies. Cellobiohydrolase is an important enzyme for degrading cellulose into cellobiose. A recent study on GH 6 cellobiohydrolase from salt marsh bacteria reported that the enzyme retained up to 87% activity in seawater, suggesting potential for application in seawater-based biorefinery (Leadbeater and Bruce 2024). Thus, the newly discovered cellobiohydrolase genes can serve as reference templates for future cloning and enzymatic characterisation.

Among the examined lignocellulolytic enzymes, hemicellulases were represented by a diverse array of GH and CE families. This reflects the structural complexity of hemicellulosic polymers. The GH families are responsible for cleaving α-(1,2)- and β-(1,3;1,4)-glycosidic bond linkages present in hemicellulose side chains and backbones (de Souza and Kawaguti 2021). Furthermore, the CE families hydrolyse ester linkage between acetyl side chain and lignin from hemicellulose backbone (Puchart and Biely 2023). The presence of side branches xylan in mangrove trees contribute to structural integrity and resistance to degradation by natural forces (Alves et al. 2020). This is significant because hemicellulose, particularly acetylated xylan, forms lignin-carbohydrates complexes that act as a protective barrier, preventing cellulose from enzymatic degradation (Curry et al. 2023). The removal of acetyl group and lignin side branches is crucial as these structural features can obstruct the activity of other GH enzymes during cellulose degradation (Nargotra et al. 2023). Notably, the Pseudomonadota phylum was identified as the major degrader for carbohydrate ester, with CE 4 as the key enzyme. In this study, CE 4 was identified affiliated with an underexplored bacteria genus, *Pseudolabrys* from the Pseudomonadota phylum. Currently, only one species was reported under the genus *Pseudolabrys* (Kämpfer et al. 2006). The lignocellulolytic abilities in this genus remain unknown. These findings suggest that *Pseudolabrys* may play an active role in carbohydrate ester degradation and require further exploration.

Among the three components of lignocellulose, lignin is the most recalcitrant polymers due to its complex and phenolic structure (Iram et al. 2021). The decomposition of lignin requires two main groups of lignin-modifying enzymes: ligninolytic enzymes and auxiliary enzymes that aid in the decomposition of lignin. Both enzyme groups were identified in this study and prominently represented by the Pseudomonadota phylum. Within the AA families, auxiliary enzymes from AA 3, 4, 6, and 7 are the main lignin-modifying enzymes, followed by the ligninolytic enzymes AA 2 family. These enzymes facilitate the oxidative reactions that disrupt the lignin structure and generating reactive intermediate to support subsequent depolymerization process of lignocellulose (Ji et al. 2024). The high abundance of auxiliary enzymes indicate that mangrove bacteria actively participate in preventing repolymerization of phenoxy radical and producing hydrogen peroxide for ligninolytic enzymes (Zhao et al. 2022). The Bacteroidota phylum that was recorded with the least amounts in lignin-modifying enzymes aligns with previous findings which reported that genes related to ligninolytic function were mostly found in Pseudomonadota, and less common in Bacteriodota (Grgas et al. 2023). Although previous metagenomic studies have highlighted carbohydrate-active enzymes involved in the degradation of hemicellulose, cellulose, and pectin, CAZy genes in the AA families were rarely discussed in their studies (Ghose et al. 2024; Paixão et al. 2021; Priya et al. 2018; Zhang et al. 2023; Zhao et al. 2019). Therefore, these findings provide an important insight into the discovery of new ligninolytic enzymes from mangrove bacteria, imposing further investigation to evaluate their potential for industrial applications.

The discovery of enzymes through functional metagenomics followed by biochemical characterisation plays a crucial role in linking genetic potential to practical industrial applications (Jeilu et al. 2024). While GH 3 β-glucosidase dominated the dataset, BGL3_GH1 from the GH 1 family was selected for functional characterisation because it is originated from the underexplored bacterial lineage. Moreover, GH 1 β-glucosidases are frequently reported to exhibit greater glucose tolerance and reduced end-product inhibition compared to the GH 3 enzymes, highlighting their greater relevance for industrial applications (Huang et al. 2023; Sun et al. 2024). Recombinant BGL3_GH1 exhibited optimal activity at pH 7.0 and 60 ℃, with substantial activity retained across neutral to mild alkaline pH. Functional assays demonstrated that recombinant BGL3_GH1 exhibited considerable enzymatic activity and stability within a pH range of 7 to 9, indicating suitability for processes operating at neutral to mild alkaline condition. Notably, its activity was enhanced in the presence of non-ionic surfactants (Tween-20, Tween-40, Tween-80, and Triton X-100) further supports its industrial potential. These surfactants are used during the pre-treatment of lignocellulosic biomass to improve enzyme accessibility (Muñoz et al. 2022).

Recombinant BGL3_GH1 retained high catalytic activity across a wide temperature range, with optimal activity at 60 ℃. However, thermostability assays revealed reduced residual activity in pre-incubation above 45 ℃ in the absence of substrate, highlighting the differences between optimal catalytic temperature and thermal stability. Previous studies have showed that ligand binding can enhance thermal stability of enzyme by increasing its conformational rigidity and resistance to thermal denaturation (Gonzalez and Sreelatha 2024; Sampson et al. 2021). While the substrate-induce stabilization was not examined in the present study, the observed activity profile suggests that this mechanism may contribute to the optimal activity of recombinant BGL3_GH1 at 60 ℃. However, further investigation will be required to assess the ligand-assisted thermostability. Acid and alkaline solution are commonly used as solvent in the pre-treatment of lignocellulosic biomass (Awogbemi and Von Kallon 2022). Upon neutralization, these solutions formed salts as byproducts and require an extra washing step to remove them. In this study, recombinant BGL3_GH1 exhibited tolerance to elevated NaCl concentration and retained high relative activity across the tested range. This characteristic is advantageous for biomass conversion processes that involved salt-generating pre-treatments, as it could reduce the requirement for extensive washing steps prior to enzymatic hydrolysis.

In addition, recombinant BGL3_GH1 also exhibited high tolerance to glucose, maintaining up to 90% activity at glucose concentration up to 3 M. In contrast, other reported glucose-tolerant GH 1 β-glucosidases retained only approximately 60% of their activity at high glucose concentration, such as 3 M for LQBG8 or 1 M for BgAr and ThBG1 (Huang et al. 2023; Sun et al. 2024; Yin et al. 2021). The glucose-enhanced activity observed for BGL3_GH1 is notable, as most GH 1 β-glucosidase are strongly inhibited by glucose. Although the underlying mechanism remains unclear, this behavior may due to that the presence of a secondary glucose-binding site on the enzyme. The binding of glucose on the secondary site may indirectly enhance catalytic activity rather than exerting an inhibitory effect (Yang et al. 2015). Notably, the high glucose tolerance observed in recombinant BGL3_GH1 underscores its potential for application in glucose-rich industrial processes. As the accumulation of glucose often leads to feedback inhibition of β-glucosidase which impaired cellulose hydrolysis (Sengupta et al. 2023). Notably, recombinant BGL3_GH1 exhibits apparent substrate affinity (2.16 mM) and catalytic efficiency (127.76 s^−1^ mM^−1^), which are comparable to a reported thermophilic GH 1 β-glucosidase (BglAc) with substrate affinity (3.41 mM) and catalytic efficiency (122.7 s^−1^ mM^−1^) (He et al. 2024). This suggests that recombinant BGL3_GH1 is functionally active at elevated temperatures and capable of hydrolyzing β-glucosidic substrates. The functional relevance of recombinant BGL3_GH1 was further demonstrated using sugarcane bagasse in a two-step saccharification assay. Supplementation with recombinant BGL3_GH1 improved the saccharification efficiency of sugarcane bagasse by 21% compared with commercial cellulase alone, indicating a clear synergistic effect. A similar trend was observed in another study that supplementation with β-glucosidase resulted in approximately sixfold increase in saccharification yield (Qu et al. 2017). The higher improvement observed in that study is likely due to longer hydrolysis durations and higher enzyme loadings. Future work will focus on optimizing reaction conditions to further enhance saccharification efficiency. Collectively, these findings suggest that recombinant BGL3_GH1 is a promising candidate for various biotechnological applications, particularly in the biofuel industry.

## Conclusion

In conclusion, this work provides the functional profile of mangrove soil bacteria, highlighting their synergistic roles in lignocellulose degradation as revealed through a shotgun metagenomic approach. The findings offer valuable insights into the bacterial consortia responsible for the degradation of lignocellulosic compounds in the mangrove ecosystems. A curated set of genes encoding enzymes involved in the degradation of cellulose, hemicellulose, and lignin was identified and bacteria from five different phyla were shown to collaborate in a synergistic network that degrades lignocellulose into sugar molecules. Notably, a large proportion of the lignocellulolytic CAZy genes were from affiliated with underexplored bacteria lineages. Such findings underscore the untapped lignocellulolytic potential of mangrove-associated bacteria. To experimentally validate the metagenomic prediction, a β-glucosidase gene, *BGL3_GH1* derived from an underexplored bacteria was cloned, expressed, and biochemically characterized. The recombinant enzyme exhibited maximum activity at 60 ℃ and pH 7.0, retained substantial activity at high salinity conditions, enhanced activity in the presence of non-ionic surfactants, and demonstrated tolerance to high glucose concentrations. Supplementation of recombinant BGL3_GH1 in the hydrolysis of sugarcane bagasse improved the saccharification yield, showing its complementary role in biomass saccharification. These favorable properties underscore its potential for industrial application in biomass degradation processes. In short, the identified 148 lignocellulolytic genes represent a valuable genetic resource for future biotechnological exploration.

### Accession numbers

The data that supports the findings of this study are available in this article, or from the corresponding author on request. The sequencing data have been deposited in the NCBI Sequence Read Archive (SRA) under BioProject accession numbers PRJNA939948. The nucleotide and protein sequence data of BGL3_GH1 were deposited in the NCBI database under accession number PX852031.

## Supplementary Information

Below is the link to the electronic supplementary material.

Supplementary Material 1

## Data Availability

The data that supports the findings of this study are available in this article, or from the corresponding author on request. The sequencing data have been deposited in the NCBI Sequence Read Archive (SRA) under BioProject accession numbers PRJNA939948. The nucleotide and protein sequence data of BGL3_GH1 were deposited in the NCBI database under accession number PX852031.

## Abbreviations

AA: Auxiliary activity
CAZy: Carbohydrate-active enzyme database
CAZymes: Carbohydrate-active enzymes
CBM: Carbohydrate-binding module
CE: Carbohydrate esterase
CMC: Carboxymethylcellulose
EDTA: Ethylenediaminetetraacetic acid
eggNOG: Evolutionary genealogy of genes: non-supervised orthologous groups
GH: Glycosyl hydrolase
GT: Glycosyl transferase
KEGG: Kyoto encyclopedia of genes and genomes
LPMO: Lytic polysaccharide monooxygenase
MAG: Metagenome-assembled genome
NCBI: National center for biotechnology information
ORFs: Open reading frames
PL: Polysaccharide lyase
SRA: Sequence read archive
SDS: Sodium dodecyl sulfate
