# Supplementary material for: Metagenomic insights into mangrove lignocellulolytic bacteria and functional analysis of a glucose-tolerant GH 1 β-glucosidase
Source: 3 Biotech. 2026 Apr 15;16(5):163. doi: 10.1007/s13205-026-04788-x (PMC13083753; doi:10.1007/s13205-026-04788-x)
Supplement: Supplementary file 1 — Supplementary Material 1 [file 13205_2026_4788_MOESM1_ESM.docx]

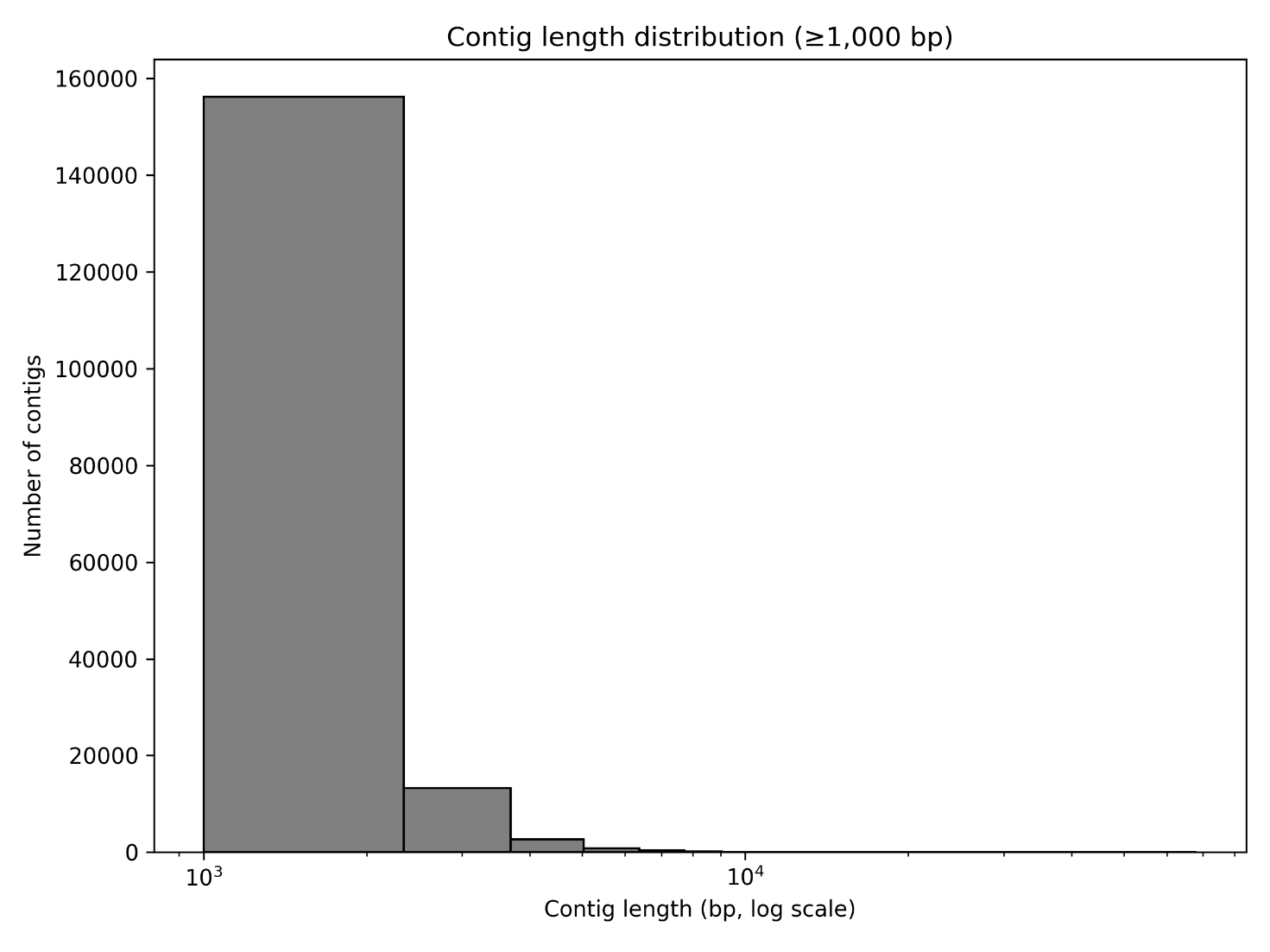
**Fig. S1**  Contig length distribution of the assembled metagenome after quality filtering. The histogram represents the length distribution of assembled contigs retained for downstream analysis.


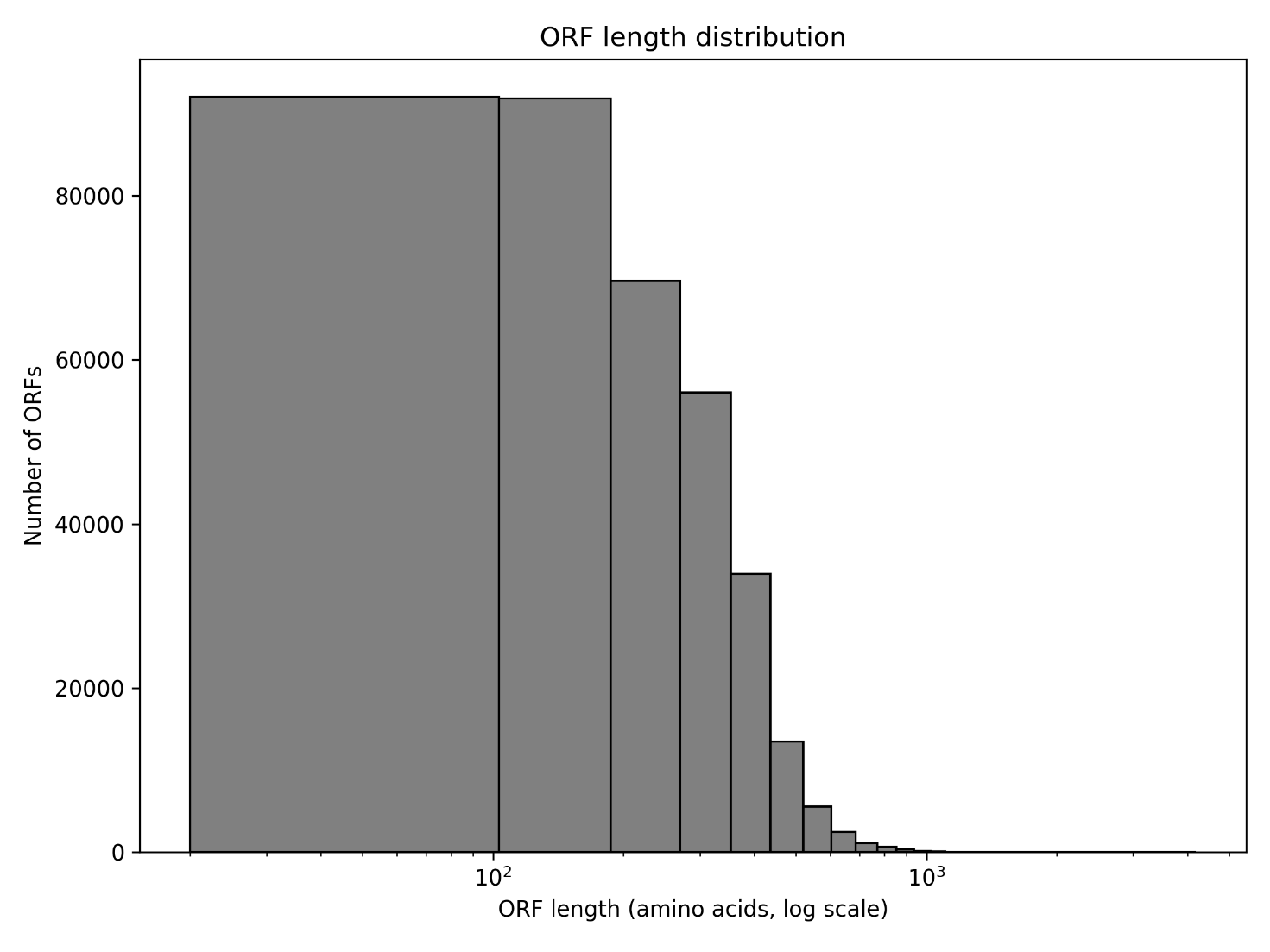


**Fig. S2**  Length distribution of predicted open reading frames (ORFs). The histogram represents the distribution of ORF lengths (in amino acids) predicted from contigs ≥ 1,000 bp using Prodigal in metagenome mode.


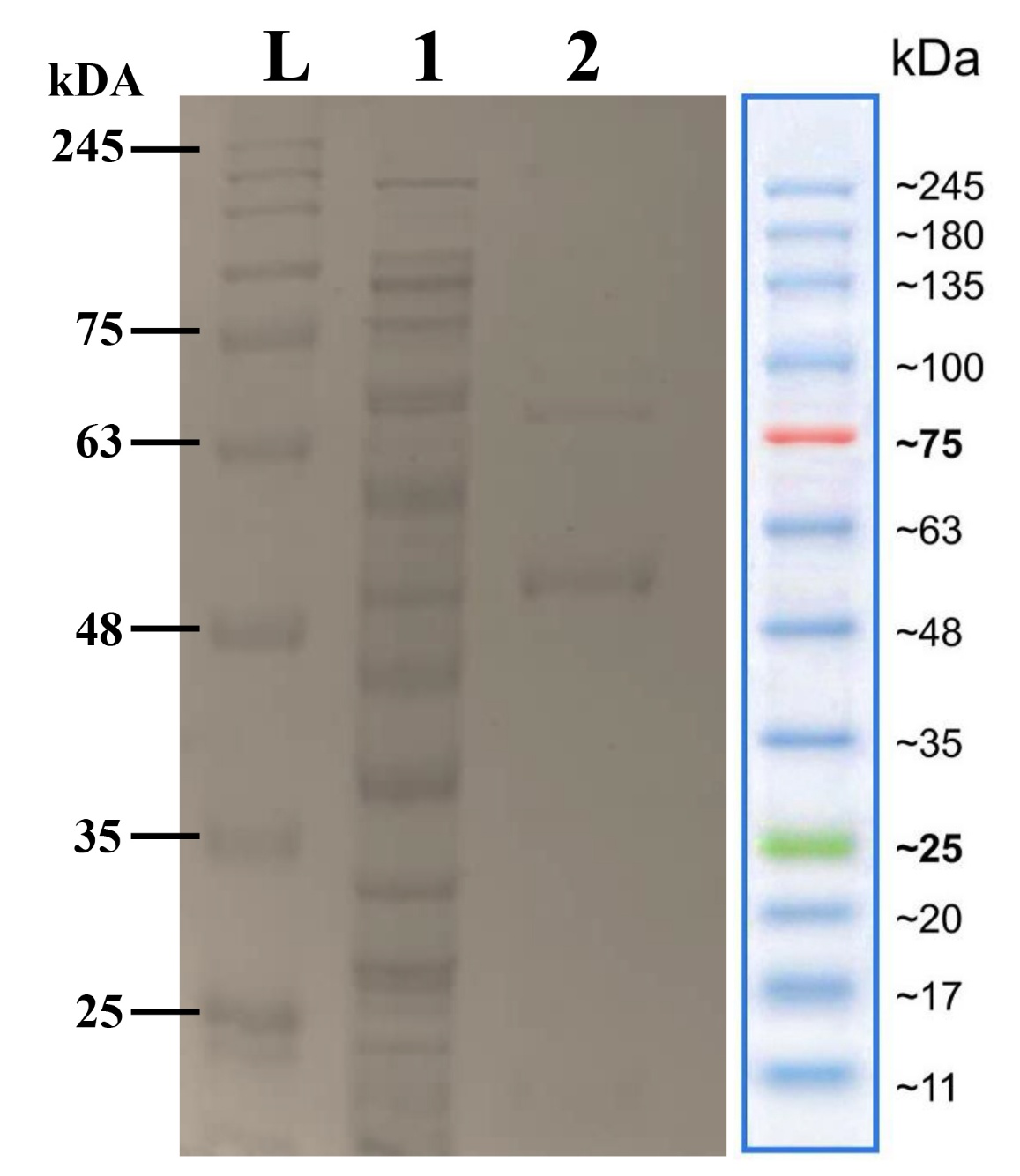


**Fig. S3** Protein size estimation of purified BGL3_GH1 using SDS-PAGE. Lane 1: Simply BlUeye Prestained Protein Ladder (Sigma-Aldrich, USA), lane 2: crude enzyme extracts, lane 3: purified BGL-3 by affinity chromatography column.


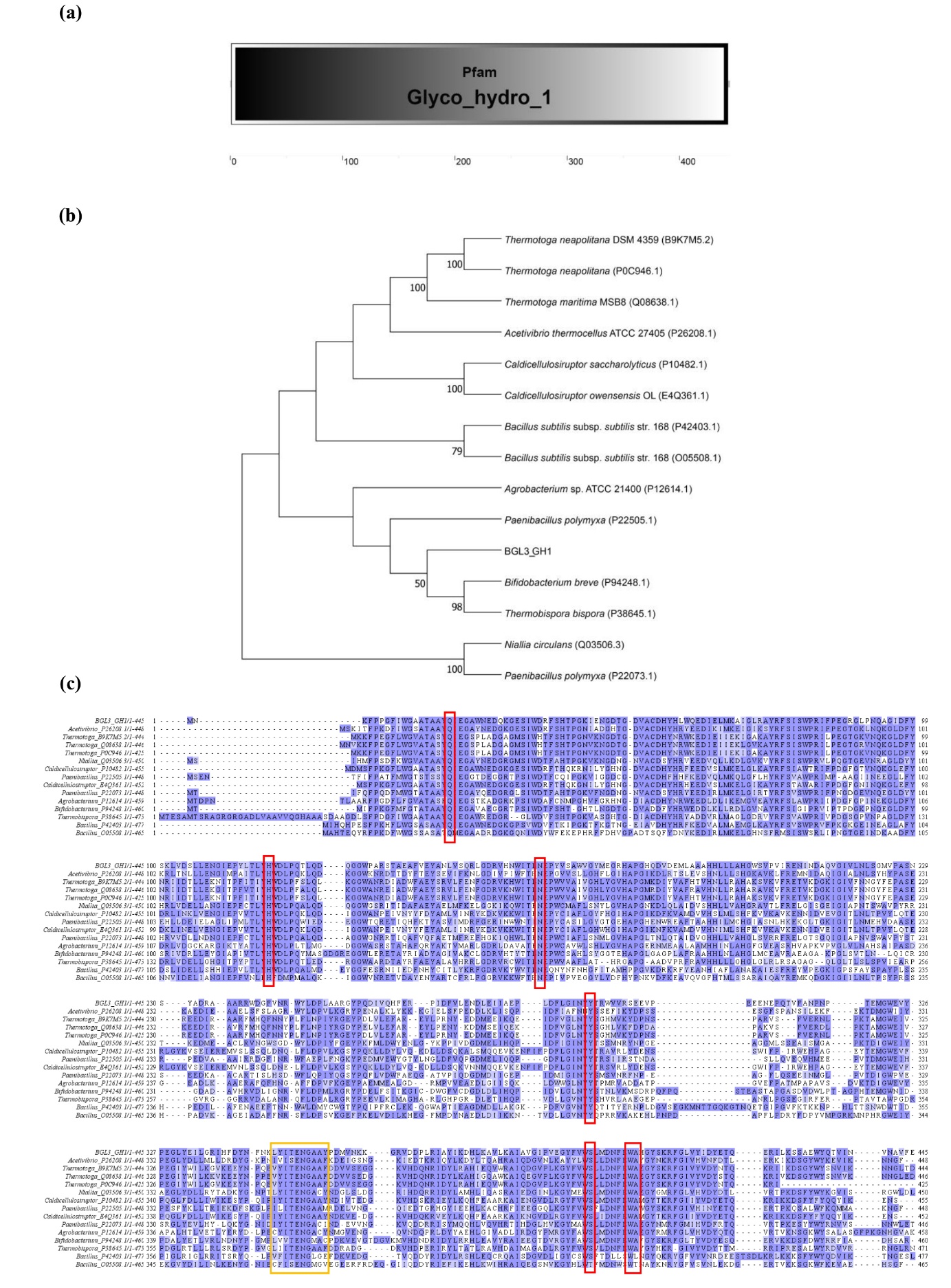


**Fig. S4** Pfam domain organization, phylogenetic analysis, and multiple sequence alignment of BGL3_GH1 with GH1 β-glucosidase from UniProtKB database. (a) Pfam domain analysis of BGL3_GH1. (b) Bootstrap consensus phylogenetic tree of protein BGL3_GH1 constructed using Neighbor-Joining method in MEGA 11. The consensus tree was generated from 1,000 bootstrap replicates, and the percentages are indicated at the nodes. (c) Multiple sequence alignment of BGL3_GH1 and selected GH1 β-glucosidases. Conserved regions are shaded according to sequence identity, the active site is indicated by orange boxes, and residue binding sites are highlighted by red boxes.


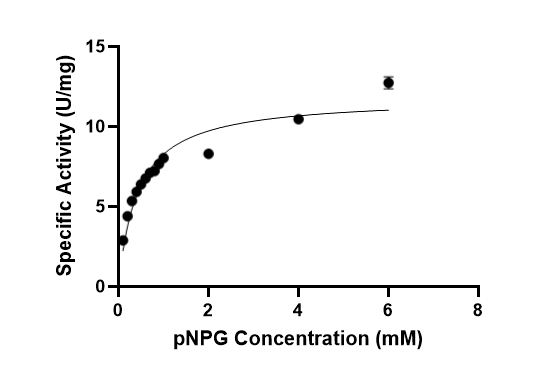


**Fig. S5** Michaelis-Menten plot of recombinant BGL3_GH1 activity toward *p*NPG. Data points represent mean initial reaction rates plotted against substrate concentration. The solid line represents non-linear regression to the Michaelis-Menten equation. Kinetic parameters and their 95 % confidence intervals were derived from pooled replicate data (n = 60).

**Table S1** Lignocellulolytic CAZymes identified from contigs ≥ 1,000 bp.

| Gene ID | Enzyme function | CAZy family | Percentage identity (%) | Query coverage (%) | Taxonomy |
| --- | --- | --- | --- | --- | --- |
| k141_1021418_436_1806_- | β-glucosidase | GH 1 | 67.3 | 93.9 | *Pseudolabrys* sp. |
| k141_966241_647_1990_- | β-glucosidase | GH 1 | 67.3 | 95.7 | *Pseudolabrys* sp. |
| k141_676226_1_1191_+ | β-glucosidase | GH 1 | 67.4 | 99.5 | *Acidimicrobiia bacterium* |
| k141_2064572_788_2125_- | β-glucosidase | GH 1 | 74.2 | 99.3 | *Anaerolineales bacterium* |
| k141_1083124_3_932_+ | β-glucosidase | GH 1 | 76.1 | 99 | *Anaerolineales bacterium* |
| k141_147756_3_1157_+ | α-glucuronidase | GH 2 | 72.2 | 99.2 | *Anaerolineales bacterium* |
| k141_969748_3316_5160_+ | α-glucuronidase | GH 2 | 63.4 | 94.3 | *Acidobacteria bacterium* |
| k141_511464_69_1802_+ | α-glucuronidase | GH 2 | 71 | 99.7 | *Thermoflexales bacterium* |
| k141_1562770_1_1818_+ | β-glucosidase | GH 3 | 59.2 | 99.5 | *Candidatus Solibacter* sp. |
| k141_1374196_3_1523_- | β-glucosidase | GH 3 | 72.4 | 98.4 | *Acidobacteria bacterium* |
| k141_1168716_108_1211_- | β-glucosidase | GH 3 | 56.8 | 99.5 | *Streptosporangiales bacterium* |
| k141_433882_1_903_- | β-glucosidase | GH 3 | 76.3 | 99.7 | *Anaerolineae bacterium* |
| k141_1187086_1_756_+ | β-glucosidase | GH 3 | 76.1 | 98.8 | *Anaerolineales bacterium* |

Table S1 Lignocellulolytic CAZymes identified from contigs ≥ 1,000 bp. (Continued)

| Gene ID | Enzyme function | CAZy family | Percentage identity (%) | Query coverage (%) | Taxonomy |
| --- | --- | --- | --- | --- | --- |
| k141_1674238_2_1795_+ | β-glucosidase | GH 3 | 70.8 | 89.3 | *Chloroflexi bacterium* |
| k141_615641_1_1503_+ | β-glucosidase | GH 3 | 87.2 | 100 | *Gemmatimonadales bacterium* |
| k141_600957_3_1490_+ | β-glucosidase | GH 3 | 73.4 | 100 | *Aurantiacibacter spongiae* |
| k141_1498217_1_1398_+ | β-glucosidase | GH 3 | 73.4 | 99.6 | *Kiloniellaceae bacterium* |
| k141_2280771_3332_5155_- | β-glucosidase | GH 3 | 75.5 | 99.8 | *Kiloniellaceae bacterium* |
| k141_1125939_662_2935_- | β-glucosidase | GH 3 | 74.1 | 99.9 | *Kiloniellaceae bacterium* |
| k141_516924_107_1174_- | Endoglucanase | GH 5 | 64 | 96.3 | *Deltaproteobacteria* |
| k141_1102371_3_1112_- | Endoglucanase | GH 5 | 67.6 | 98.4 | *Paraglaciecola polaris* |
| k141_611854_99_1127_- | Endoglucanase | GH 5 | 68.5 | 93.3 | *Anaerolineae* |
| k141_1841314_1_1134_+ | Cellobiohydrolase | GH 6 | 69.6 | 62.4 | *Streptosporangiales bacterium* |
| k141_1306646_317_1084_- | Endo-1,4-beta-xylanase | GH 10 | 84.6 | 99.2 | *Anaerolineales bacterium* |

Table S1 Lignocellulolytic CAZymes identified from contigs ≥ 1,000 bp. (Continued)

| Gene ID | Enzyme function | CAZy family | Percentage identity (%) | Query coverage (%) | Taxonomy |
| --- | --- | --- | --- | --- | --- |
| k141_1484674_1_933_- | Endo-1,4-beta-xylanase | GH 10 | 54.9 | 91.3 | *Pyrinomonadaceae bacterium* |
| k141_756197_918_1667_+ | Endo-1,4-beta-xylanase | GH 10 | 92 | 99.6 | *Anaerolineales bacterium* |
| k141_586564_582_1742_+ | Endo-1,4-beta-xylanase | GH 10 | 71.3 | 96.9 | *Deltaproteobacteria bacterium* |
| k141_483728_1092_2738_+ | β-xylosidase | GH 39 | 71.8 | 95.6 | *Acidobacteria bacterium* |
| k141_689775_3_653_+ | α-arabinofuranosidase | GH 51 | 78.6 | 99.5 | *Acidobacteria bacterium* |
| k141_833653_573_1520_- | α-arabinofuranosidase | GH 51 | 78.7 | 99.7 | *Acidobacteriia bacterium* |
| k141_761142_1078_2517_- | α-arabinofuranosidase | GH 51 | 80.1 | 99.8 | *Luteitalea* sp. |

Table S1 Lignocellulolytic CAZymes identified from contigs ≥ 1,000 bp. (Continued)

| Gene ID | Enzyme function | CAZy family | Percentage identity (%) | Query coverage (%) | Taxonomy |
| --- | --- | --- | --- | --- | --- |
| k141_996708_477_1241_- | α-arabinofuranosidase | GH 51 | 64.3 | 98 | *Firmicutes bacterium* |
| k141_1301715_215_1369_- | α-arabinofuranosidase | GH 51 | 76.3 | 100 | *Bacteroides* sp. |
| k141_1608819_1_798_- | α-arabinofuranosidase | GH 51 | 82.7 | 100 | *Bacteroides* sp. |
| k141_2570953_171_1346_- | α-arabinofuranosidase | GH 51 | 73.9 | 100 | *Chloroflexi bacterium* |
| k141_2178810_1_1005_- | α-arabinofuranosidase | GH 51 | 74.9 | 99.7 | *Chloroflexi bacterium* |
| k141_1577447_2_1060_- | α-arabinofuranosidase | GH 51 | 86.7 | 100 | *Chloroflexi bacterium* |
| k141_2220318_1_1113_- | α-arabinofuranosidase | GH 51 | 82.5 | 100 | *Ardenticatenaceae bacterium* |

Table S1 Lignocellulolytic CAZymes identified from contigs ≥ 1,000 bp. (Continued)

| Gene ID | Enzyme function | CAZy family | Percentage identity (%) | Query coverage (%) | Taxonomy |
| --- | --- | --- | --- | --- | --- |
| k141_678669_214_1359_- | α-arabinofuranosidase | GH 51 | 69.7 | 99.5 | *Gammaproteobacteria bacterium* |
| k141_2352041_138_1388_+ | arabinogalactan endo-1,4-beta-galactosidase | GH 53 | 61.7 | 99 | *Anaerolineaceae bacterium* |
| k141_324241_3_965_- | arabinogalactan endo-1,4-beta-galactosidase | GH 53 | 58.7 | 98.8 | *Trinickia* sp. |
| k141_1824751_3_2081_- | arabinogalactan endo-1,4-beta-galactosidase | GH 67 | 71.2 | 99.1 | *Acidobacteria bacterium* |
| k141_203839_1_1176_+ | α-glucuronidase | GH 67 | 86.6 | 97.2 | *Acidobacteria bacterium* |
| k141_1331631_1_1068_- | α-glucuronidase | GH 67 | 78.4 | 100 | *Anaerolineales bacterium* |
| k141_1930122_94_1212_- | α-glucuronidase | GH 67 | 78.4 | 98.4 | *Anaerolineae bacterium* |

Table S1 Lignocellulolytic CAZymes identified from contigs ≥ 1,000 bp. (Continued)

| Gene ID | Enzyme function | CAZy family | Percentage identity (%) | Query coverage (%) | Taxonomy |
| --- | --- | --- | --- | --- | --- |
| k141_2112347_1_1038_+ | α-glucuronidase | GH 67 | 79.4 | 98.6 | *Anaerolineae bacterium* |
| k141_2037482_725_2083_+ | α-glucuronidase | GH 67 | 74.6 | 100 | *Anaerolineae bacterium* |
| k141_1586402_3_2162_- | α-glucuronidase | GH 67 | 72 | 98.7 | *Sphingomonas* sp. |
| k141_2522900_1_894_- | Acetyl xylan esterase | CE 1 | 76.2 | 98 | *Mycolicibacterium confluentis* |
| k141_1445615_3_629_- | Acetyl xylan esterase | CE 1 | 82.3 | 100 | *Mycobacterium* sp. |
| k141_2082441_3_1073_+ | Acetyl xylan esterase | CE 1 | 82.2 | 99.2 | *Prolixibacteraceae bacterium* |
| k141_290589_41_1123_- | Acetyl xylan esterase | CE 1 | 87.8 | 100 | *Anaerolineae bacterium* |
| k141_2154138_620_1753_+ | Acetyl xylan esterase | CE 1 | 82.2 | 100 | *Anaerolineae bacterium* |

Table S1 Lignocellulolytic CAZymes identified from contigs ≥ 1,000 bp. (Continued)

| Gene ID | Enzyme function | CAZy family | Percentage identity (%) | Query coverage (%) | Taxonomy |
| --- | --- | --- | --- | --- | --- |
| k141_543680_2_1027_+ | Acetyl xylan esterase | CE 1 | 91.8 | 99.7 | *Gemmatimonadetes bacterium* |
| k141_1332410_620_2740_- | Acetyl xylan esterase | CE 1 | 63.5 | 52 | *Aquisphaera giovannonii* |
| k141_1727861_183_1403_- | Acetyl xylan esterase | CE 1 | 67.6 | 96.3 | *Verrucomicrobia bacterium* |
| k141_1418885_340_1242_- | Polysaccharide deacetylase | CE 4 | 66.7 | 79.7 | *Pseudolabrys* sp. |
| k141_347886_480_1253_+ | Polysaccharide deacetylase | CE 4 | 75.9 | 98.4 | *Hyphomicrobiales bacterium* |
| k141_2166720_444_1550_+ | Polysaccharide deacetylase | CE 4 | 70.4 | 99.2 | *Hyphomicrobiaceae bacterium* |
| k141_435618_572_1462_+ | Polysaccharide deacetylase | CE 4 | 74.3 | 100 | *Proteobacteria bacterium* |

Table S1 Lignocellulolytic CAZymes identified from contigs ≥ 1,000 bp. (Continued)

| Gene ID | Enzyme function | CAZy family | Percentage identity (%) | Query coverage (%) | Taxonomy |
| --- | --- | --- | --- | --- | --- |
| k141_1227744_553_1452_- | Polysaccharide deacetylase | CE 4 | 71 | 98.7 | *Pseudolabrys* sp. |
| k141_1609394_202_1053_+ | Acetyl xylan esterase | CE 6 | 85.9 | 100 | *Acidobacteria bacterium* |
| k141_43042_1_693_+ | Acetyl xylan esterase | CE 7 | 73.9 | 100 | *Paenibacillus* sp. |
| k141_1052167_273_1241_+ | Acetyl xylan esterase | CE 7 | 79.4 | 99.7 | *Anaerolineales bacterium* |
| k141_1584997_1_678_- | Acetyl xylan esterase | CE 7 | 86.7 | 100 | *Anaerolineae bacterium* |
| k141_878373_1_831_- | Acetyl xylan esterase | CE 15 | 64 | 99.3 | *Planctomycetes bacterium* |
| k141_2499788_2_769_- | Catalase-peroxidase | AA 2 | 81.6 | 100 | *Acidimicrobiales bacterium* |

Table S1 Lignocellulolytic CAZymes identified from contigs ≥ 1,000 bp. (Continued)

| Gene ID | Enzyme function | CAZy family | Percentage identity (%) | Query coverage (%) | Taxonomy |
| --- | --- | --- | --- | --- | --- |
| k141_2165109_1_1089_+ | Catalase-peroxidase | AA 2 | 85.1 | 100 | *Solirubrobacterales bacterium* |
| k141_1428906_3_905_- | Catalase-peroxidase | AA 2 | 89.1 | 97.7 | *Bacteroidetes bacterium* |
| k141_606513_1_1467_- | Catalase-peroxidase | AA 2 | 78.8 | 100 | *Anaerolinea* sp. |
| k141_989650_1_1008_- | Catalase-peroxidase | AA 2 | 90.2 | 100 | *Chloroflexi bacterium* |
| k141_1459823_3_1514_- | Catalase-peroxidase | AA 2 | 87.7 | 100 | *Chloroflexi bacterium* |
| k141_2276449_2_1636_- | Catalase-peroxidase | AA 2 | 82.6 | 99.3 | *Phycisphaerae bacterium* |
| k141_1142439_2_1555_+ | Catalase-peroxidase | AA 2 | 90 | 100 | *Candidatus Poribacteria bacterium* |
| k141_1007224_1_1494_+ | Catalase-peroxidase | AA 2 | 83.9 | 99.8 | *Bauldia* sp. |
| k141_1682440_3_1637_+ | Catalase-peroxidase | AA 2 | 84.3 | 99.8 | *Methyloligella* sp. |
| k141_2245337_77_2038_- | Catalase-peroxidase | AA 2 | 85 | 99.8 | *Methyloligella* sp. |
| k141_304285_2_1099_+ | Catalase-peroxidase | AA 2 | 92.1 | 100 | *Thiohalobacter* sp. |
| k141_443275_2_1153_- | Catalase-peroxidase | AA 2 | 89.3 | 100 | *Thiohalocapsa* sp. |
| k141_2490356_2_1174_+ | Catalase-peroxidase | AA 2 | 88.4 | 99.7 | *Acidithiobacillales bacterium* |

Table S1 Lignocellulolytic CAZymes identified from contigs ≥ 1,000 bp. (Continued)

| Gene ID | Enzyme function | CAZy family | Percentage identity (%) | Query coverage (%) | Taxonomy |
| --- | --- | --- | --- | --- | --- |
| k141_1942192_2_1945_+ | Catalase-peroxidase | AA 2 | 90.9 | 99.8 | *Acidithiobacillales bacterium* |
| k141_903021_1_1398_- | Catalase-peroxidase | AA 2 | 89.9 | 100 | *Deltaproteobacteria bacterium* |
| k141_1073307_2_1102_+ | Catalase-peroxidase | AA 2 | 88.6 | 100 | *Deltaproteobacteria bacterium* |
| k141_738562_2_1093_+ | Catalase-peroxidase | AA 2 | 92.3 | 100 | *Deltaproteobacteria bacterium* |
| k141_910796_220_1701_+ | Catalase-peroxidase | AA 2 | 87.2 | 99.8 | *Deltaproteobacteria bacterium* |
| k141_1586659_48_1037_- | Catalase-peroxidase | AA 2 | 84.1 | 99.4 | *Deltaproteobacteria bacterium* |
| k141_352750_1444_2460_- | Catalase-peroxidase | AA 2 | 89.9 | 99.4 | *Deltaproteobacteria bacterium* |
| k141_1771162_1_1038_+ | Catalase-peroxidase | AA 2 | 90.4 | 100 | *Deltaproteobacteria bacterium* |
| k141_1862212_2_1087_+ | Catalase-peroxidase | AA 2 | 88.4 | 100 | *Deltaproteobacteria bacterium* |
| k141_1413882_1_1101_- | Catalase-peroxidase | AA 2 | 92.3 | 88.3 | *Gammaproteobacteria bacterium* |
| k141_1822738_3_1076_+ | Catalase-peroxidase | AA 2 | 90.8 | 100 | *Gammaproteobacteria bacterium* |
| k141_1078341_2_1018_+ | Catalase-peroxidase | AA 2 | 89.8 | 98.2 | *Candidatus Rokubacteria bacterium* |
| k141_534929_1233_2180_+ | Catalase-peroxidase | AA 2 | 91.5 | 100 | *Desulfofustis* sp. |

Table S1 Lignocellulolytic CAZymes identified from contigs ≥ 1,000 bp. (Continued)

| Gene ID | Enzyme function | CAZy family | Percentage identity (%) | Query coverage (%) | Taxonomy |
| --- | --- | --- | --- | --- | --- |
| k141_520745_3_1109_+ | Catalase-peroxidase | AA 2 | 85.1 | 100 | *Desulfobacterales bacterium* |
| k141_1434497_1_1392_- | Catalase-peroxidase | AA 2 | 88.4 | 100 | *Desulfobacterales bacterium* |
| k141_2381189_2450_3658_- | Catalase-peroxidase | AA 2 | 89.3 | 100 | *Desulfobacterales bacterium* |
| k141_433821_380_1957_- | GMC family oxidoreductase | AA 3 | 68.4 | 99.2 | *Mycobacterium* sp. |
| k141_702842_2_1264_- | GMC family oxidoreductase | AA 3 | 82.2 | 100 | *Hyphomicrobiales bacterium* |
| k141_2151157_108_1733_- | GMC family oxidoreductase | AA 3 | 84.7 | 100 | *Hyphomicrobiales bacterium* |
| k141_2570208_2_1258_- | GMC family oxidoreductase | AA 3 | 87.6 | 100 | *Betaproteobacteria bacterium* |
| k141_551834_3_1478_+ | GMC family oxidoreductase | AA 3 | 84.7 | 100 | *Proteobacteria bacterium* |

Table S1 Lignocellulolytic CAZymes identified from contigs ≥ 1,000 bp. (Continued)

| Gene ID | Enzyme function | CAZy family | Percentage identity (%) | Query coverage (%) | Taxonomy |
| --- | --- | --- | --- | --- | --- |
| k141_1415772_708_2369_- | GMC family oxidoreductase | AA 3 | 78.4 | 98.6 | *Hyphomicrobiaceae bacterium* |
| k141_2057124_682_2331_- | GMC family oxidoreductase | AA 3 | 74.9 | 99.5 | *Filomicrobium* sp. |
| k141_342681_2_856_+ | GMC family oxidoreductase | AA 3 | 74.3 | 98.6 | *Rhodocyclaceae bacterium* |
| k141_2141099_2_937_+ | GMC family oxidoreductase | AA 3 | 75.9 | 100 | *Betaproteobacteria bacterium* |
| k141_1685993_1_888_+ | GMC family oxidoreductase | AA 3 | 79.3 | 100 | *Gammaproteobacteria bacterium* |
| k141_1525795_2_910_+ | GMC family oxidoreductase | AA 3 | 70.1 | 98.7 | *Betaproteobacteria bacterium* |
| k141_1083283_3_1193_- | FAD-binding oxidoreductase | AA 4 | 72 | 100 | *Chloroflexi bacterium* |

Table S1 Lignocellulolytic CAZymes identified from contigs ≥ 1,000 bp. (Continued)

| Gene ID | Enzyme function | CAZy family | Percentage identity (%) | Query coverage (%) | Taxonomy |
| --- | --- | --- | --- | --- | --- |
| k141_2058635_2_1276_- | FAD-binding oxidoreductase | AA 4 | 76.7 | 100 | *Chloroflexi bacterium* |
| k141_1972714_1_906_- | FAD-binding oxidoreductase | AA 4 | 88.4 | 96.7 | *Anaerolineae bacterium* |
| k141_1631635_1_1098_- | FAD-binding oxidoreductase | AA 4 | 88.8 | 100 | *Gammaproteobacteria bacterium* |
| k141_1462128_3_812_- | FAD-binding oxidoreductase | AA 4 | 84.4 | 100 | *Alphaproteobacteria bacterium* |
| k141_272839_3_1019_- | FAD-binding oxidoreductase | AA 4 | 83.3 | 97.1 | *Pseudolabrys* sp. |
| k141_1197467_583_1374_+ | FAD-binding oxidoreductase | AA 4 | 85.1 | 99.2 | *Pseudolabrys* sp. |
| k141_1069429_2_1105_- | FAD-binding oxidoreductase | AA 4 | 78.5 | 100 | *Deltaproteobacteria bacterium* |

Table S1 Lignocellulolytic CAZymes identified from contigs ≥ 1,000 bp. (Continued)

| Gene ID | Enzyme function | CAZy family | Percentage identity (%) | Query coverage (%) | Taxonomy |
| --- | --- | --- | --- | --- | --- |
| k141_629812_2_1075_+ | FAD-binding oxidoreductase | AA 4 | 82.2 | 94.4 | *Pseudolabrys* sp. |
| k141_2351852_3_731_- | FAD-binding oxidoreductase | AA 4 | 81.4 | 99.6 | *Hyphomicrobiaceae bacterium* |
| k141_2541917_252_1613_- | FAD-binding oxidoreductase | AA 4 | 78.8 | 100 | *Gammaproteobacteria bacterium* |
| k141_1663316_1_891_- | FAD-binding oxidoreductase | AA 4 | 78.5 | 100 | *Desulfobacterales bacterium* |
| k141_1584173_75_1460_- | FAD-binding oxidoreductase | AA 4 | 78.7 | 100 | *Deltaproteobacteria bacterium* |
| k141_1019330_895_1974_+ | FAD-binding oxidoreductase | AA 4 | 82.5 | 100 | *Desulfobacterales bacterium* |
| k141_176822_1934_3358_- | FAD-binding oxidoreductase | AA 4 | 80.4 | 100 | *Hyphomicrobiales bacterium* |

Table S1 Lignocellulolytic CAZymes identified from contigs ≥ 1,000 bp. (Continued)

| Gene ID | Enzyme function | CAZy family | Percentage identity (%) | Query coverage (%) | Taxonomy |
| --- | --- | --- | --- | --- | --- |
| k141_1667678_5203_5814_- | NADPH quinone oxidoreductase | AA 6 | 91.1 | 99.5 | *Nitrospira bacterium* |
| k141_2434431_884_1495_+ | NADPH quinone oxidoreductase | AA 6 | 83.3 | 100 | *Nitrospirae bacterium* |
| k141_1747375_851_1417_- | NADPH quinone oxidoreductase | AA 6 | 97.9 | 99.5 | *Methyloceanibacter* |
| k141_2521869_401_1018_- | NADPH quinone oxidoreductase | AA 6 | 91.7 | 100 | *Methyloceanibacter* sp. |
| k141_114261_1142_1759_- | NADPH quinone oxidoreductase | AA 6 | 90.6 | 99 | *Methyloceanibacter superfactus* |
| k141_1254289_1210_1827_- | NADPH quinone oxidoreductase | AA 6 | 83.9 | 100 | *Desulfobacca acetoxidans* |
| k141_177677_1123_1449_- | NADPH quinone oxidoreductase | AA 6 | 96.3 | 99.1 | *Desulfobacterales bacterium* |

Table S1 Lignocellulolytic CAZymes identified from contigs ≥ 1,000 bp. (Continued)

| Gene ID | Enzyme function | CAZy family | Percentage identity (%) | Query coverage (%) | Taxonomy |
| --- | --- | --- | --- | --- | --- |
| k141_540130_2_325_- | NADPH quinone oxidoreductase | AA 6 | 84.3 | 100 | *Deltaproteobacteria bacterium* |
| k141_813358_3_452_- | NADPH quinone oxidoreductase | AA 6 | 92 | 100 | *Desulfobacterales bacterium* |
| k141_1018620_142_753_+ | NADPH quinone oxidoreductase | AA 6 | 89.1 | 99 | *Desulfobacterales bacterium* |
| k141_1061720_563_1174_- | NADPH quinone oxidoreductase | AA 6 | 87.6 | 99.5 | *Deltaproteobacteria bacterium* |
| k141_106434_405_1016_+ | NADPH quinone oxidoreductase | AA 6 | 89.1 | 99.5 | *Desulfobacterales bacterium* |
| k141_1322414_569_1180_- | NADPH quinone oxidoreductase | AA 6 | 86.6 | 99.5 | *Deltaproteobacteria bacterium* |
| k141_2412112_737_1342_+ | NADPH quinone oxidoreductase | AA 6 | 85.1 | 100 | *Deltaproteobacteria bacterium* |

Table S1 Lignocellulolytic CAZymes identified from contigs ≥ 1,000 bp. (Continued)

| Gene ID | Enzyme function | CAZy family | Percentage identity (%) | Query coverage (%) | Taxonomy |
| --- | --- | --- | --- | --- | --- |
| k141_805011_28_639_- | NADPH quinone oxidoreductase | AA 6 | 78.1 | 99 | *Deltaproteobacteria bacterium* |
| k141_1768596_260_871_+ | NADPH quinone oxidoreductase | AA 6 | 90.1 | 99.5 | *Desulfobacterales bacterium* |
| k141_1120426_1_222_- | NADPH quinone oxidoreductase | AA 6 | 78.4 | 100 | *Geminicoccaceae bacterium* |
| k141_2509679_705_1127_+ | NADPH quinone oxidoreductase | AA 6 | 81.6 | 100 | *Burkholderiaceae bacterium* |
| k141_2068127_195_1562_- | FAD-binding oxidoreductase | AA 7 | 88.5 | 99.6 | *Mycobacterium* sp. |
| k141_2283895_2_1207_+ | FAD-binding oxidoreductase | AA 7 | 61.3 | 99.8 | *Gemmatimonadetes bacterium* |
| k141_236104_1_657_- | FAD-binding oxidoreductase | AA 7 | 61.9 | 99.5 | *Gemmatimonadetes bacterium* |

Table S1 Lignocellulolytic CAZymes identified from contigs ≥ 1,000 bp. (Continued)

| Gene ID | Enzyme function | CAZy family | Percentage identity (%) | Query coverage (%) | Taxonomy |
| --- | --- | --- | --- | --- | --- |
| k141_2395988_921_2174_+ | FAD-binding oxidoreductase | AA 7 | 78.9 | 100 | *Planctomycetes bacterium* |
| k141_176822_431_1855_- | FAD-binding oxidoreductase | AA 7 | 78.5 | 100 | *Pseudolabrys* sp. |
| k141_1398458_1837_3261_- | FAD-binding oxidoreductase | AA 7 | 80.4 | 100 | *Pseudolabrys taiwanensis* |
| k141_252145_268_1065_+ | FAD-binding oxidoreductase | AA 7 | 69.9 | 100 | *Paraburkholderia* sp. |
| k141_903613_79_1473_- | FAD-binding oxidoreductase | AA 7 | 64.6 | 96.8 | *Paraburkholderia piptadeniae* |
